# Discovery of new cellulases from the metagenome by a metagenomics-guided strategy

**DOI:** 10.1186/s13068-016-0557-3

**Published:** 2016-07-04

**Authors:** Chao Yang, Yu Xia, Hong Qu, An-Dong Li, Ruihua Liu, Yubo Wang, Tong Zhang

**Affiliations:** Environmental Biotechnology Laboratory, The University of Hong Kong, Pokfulam Road, Pok Fu Lam, Hong Kong; State Key Laboratory of Medicinal Chemical Biology and College of Life Sciences, Nankai University, Tianjin, 300071 China; College of Life Sciences, Peking University, Beijing, 100871 China

**Keywords:** Cellulase, Glycoside hydrolase, Metagenome

## Abstract

**Background:**

Energy shortage has become a global problem. Production of biofuels from renewable biomass resources is an inevitable trend of sustainable development. Cellulose is the most abundant and renewable resource in nature. Lack of new cellulases with unique properties has become the bottleneck of the efficient utilization of cellulose. Environmental metagenomes are regarded as huge reservoirs for a variety of cellulases. However, new cellulases cannot be obtained easily by functional screening of metagenomic libraries.

**Results:**

In this work, a metagenomics-guided strategy for obtaining new cellulases from the metagenome was proposed. Metagenomic sequences of DNA extracted from the anaerobic beer lees converting consortium enriched at thermophilic conditions were assembled, and 23 glycoside hydrolase (GH) sequences affiliated with the GH family 5 were identified. Among the 23 GH sequences, three target sequences (designated as cel7482, cel3623 and cel36) showing low identity with those known GHs were chosen as the putative cellulase genes to be functionally expressed in *Escherichia coli* after PCR cloning. The three cellulases were classified into endo-β-1,4-glucanases by product pattern analysis. The recombinant cellulases were more active at pH 5.5 and within a temperature range of 60–70 °C. Computer-assisted 3D structure modeling indicated that the active residues in the active site of the recombinant cellulases were more similar to each other compared with non-active site residues. The recombinant cel7482 was extremely tolerant to 2 M NaCl, suggesting that cel7482 may be a halotolerant cellulase. Moreover, the recombinant cel7482 was shown to have an ability to resist three ionic liquids (ILs), which are widely used for cellulose pretreatment. Furthermore, active cel7482 was secreted by the twin-arginine translocation (Tat) pathway of *Bacillus subtilis* 168 into the culture medium, which facilitates the subsequent purification and reduces the formation of inclusion body in the context of overexpression.

**Conclusions:**

This study demonstrated a simple and efficient method for direct cloning of new cellulase genes from environmental metagenomes. In the future, the metagenomics-guided strategy may be applied to the high-throughput screening of new cellulases from environmental metagenomes.

**Electronic supplementary material:**

The online version of this article (doi:10.1186/s13068-016-0557-3) contains supplementary material, which is available to authorized users.

## Background

Cellulose, a renewable biopolymer composed of D-glucopyranose units linked by β-1,4-glucosidic bonds, is commonly used as raw material for the production of important industrial chemicals such as soluble sugars and biofuels [1]. Cellulases involved in hydrolyzing cellulose are composed of endoglucanases, exoglucanases and β-glucosidases. Endoglucanases (EC 3.2.1.4) hydrolyze the internal bonds randomly in the cellulose chain into cellobiose or cello-oligosaccharides. Exoglucanases (EC 3.2.1.91) release cellobiose from either the reducing or the non-reducing ends of the cellulose chain. β-Glucosidases (EC 3.2.1.21) hydrolyze cellobiose to glucose [1].

Discovery of novel enzymes through metagenomics has recently been shown to have enormous potential for obtaining a wide variety of useful biocatalysts [2, 3]. Functional metagenomics has become a routine method for the discovery of industrially relevant enzymes from natural and artificially engineered ecosystems. However, very few active clones could be obtained from huge quantities of clones tested using functional screening protocols [3].

Although some cellulase genes had been obtained from the metagenome by functional screening [4–6], the efficiency of the strategy for the discovery of new cellulases from environmental metagenomes can hardly meet the increasing industrial demand [7]. The number of new metagenome-derived cellulases could be significantly increased when novel cellulase sequences obtained by high-throughput sequencing are selected for subsequent expression.

Many researches have focused on the annotation of carbohydrate-active enzymes (CAZymes) in cellulose-degrading consortia using metagenomic analysis [8, 9]. CAZyme families play a crucial role in breakdown of complex carbohydrates. The CAZy database (http://www.cazy.org) defined 135 families of glycoside hydrolases (GHs) based on amino acid sequence similarities and structural features, which provides a powerful tool to annotate functions of obtained GH genes [10]. GHs are a widespread category of enzymes capable of cleaving the glycosidic bond in the polysaccharide chains. These GHs possess different substrate specificities, and some GH families (e.g., GH5 and GH9) were found to have cellulase activity.

**Fig. 1:**
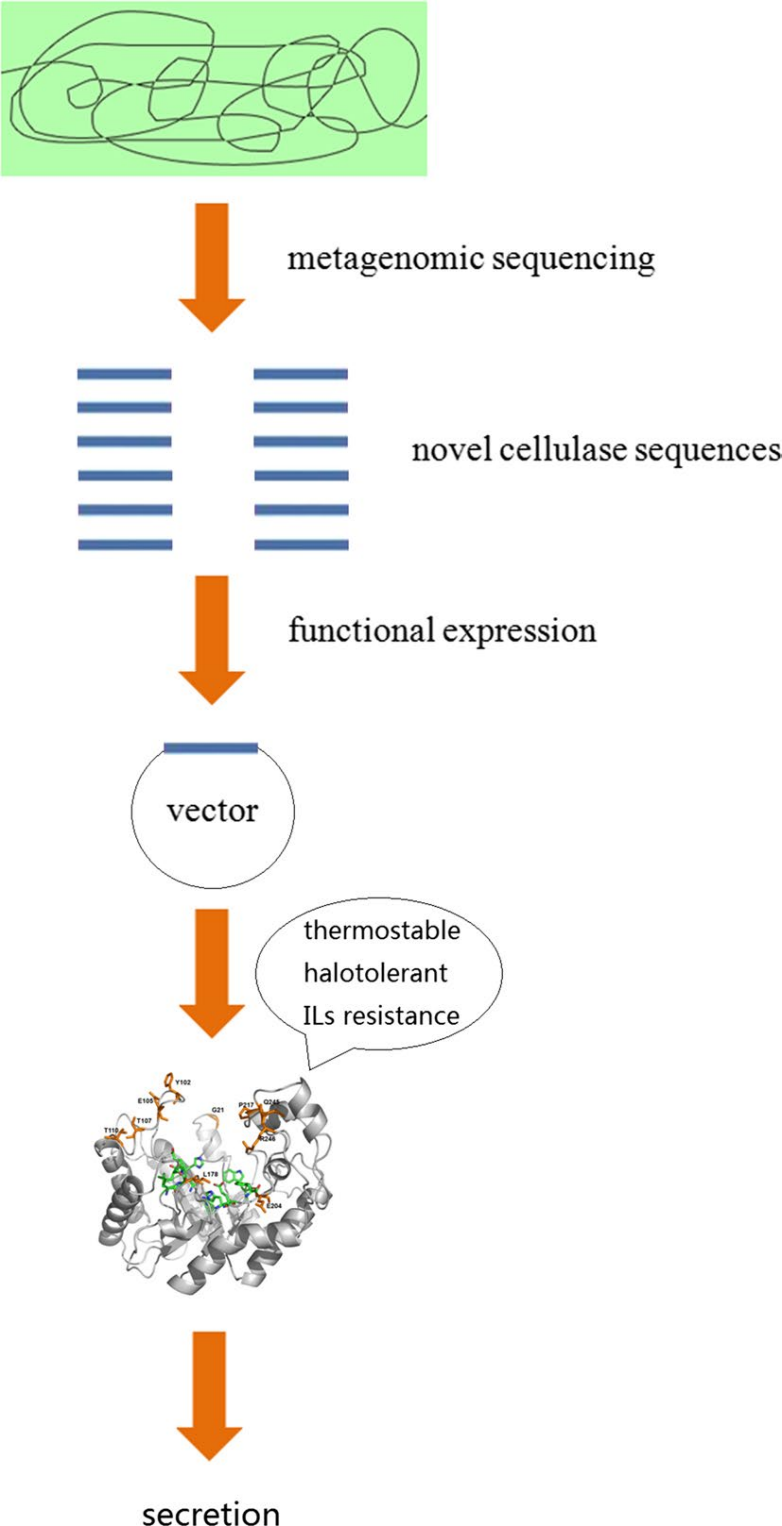
A pipeline of the metagenomics-guided strategy used in this study

It is difficult to obtain new cellulases by functional screening of metagenomic libraries because the strategy is based on cellulase activity rather than sequence similarity. Metagenomic sequencing allows targeted selection of novel cellulase sequences for high-throughput expression. In this work, a metagenomics-guided strategy was applied to clone three new cellulase genes from environmental metagenomes. Furthermore, the recombinant cellulases were functionally expressed in *Escherichia coli* and their enzymatic characteristics were investigated. A pipeline of the metagenomics-guided strategy used in this study is shown in Fig. 1.

## Results

### Cloning of three cellulase genes from the metagenome by a metagenomics-guided strategy

In this work, we have focused on the GH family 5 (GH5), mainly consisting of a variety of endoglucanases and exoglucanases that are involved in the hydrolysis of cellulose. In total, we obtained 23 GH sequences belonging to the GH5 family by metagenomic sequencing and ORFs annotation. The nucleotide sequences of 23 GHs are shown in Additional file 1: Figure S1. Among 23 GH sequences, we chose three target sequences (id_7482, 1026; id_3623, 1035; id_36, 1548 bp), which showed low similarity with those GH sequences deposited in GenBank. Using the NCBI BlastP search, id_7482 showed 54 % identity with a putative cellulase from uncultured bacterium in a laboratory biogas digester treating rice straws (GenBank accession no. AEV59734); id_3623 showed 51 % identity with an endoglucanase from *Anaerolinea thermolimosa* from sludge from a thermophilic UASB reactor (GenBank accession no. GAP08306); id_36 showed 48 % identity with a cellulase from *Acetivibrio cellulolyticus* (GenBank accession no.WP_010249757).

**Fig. 2:**
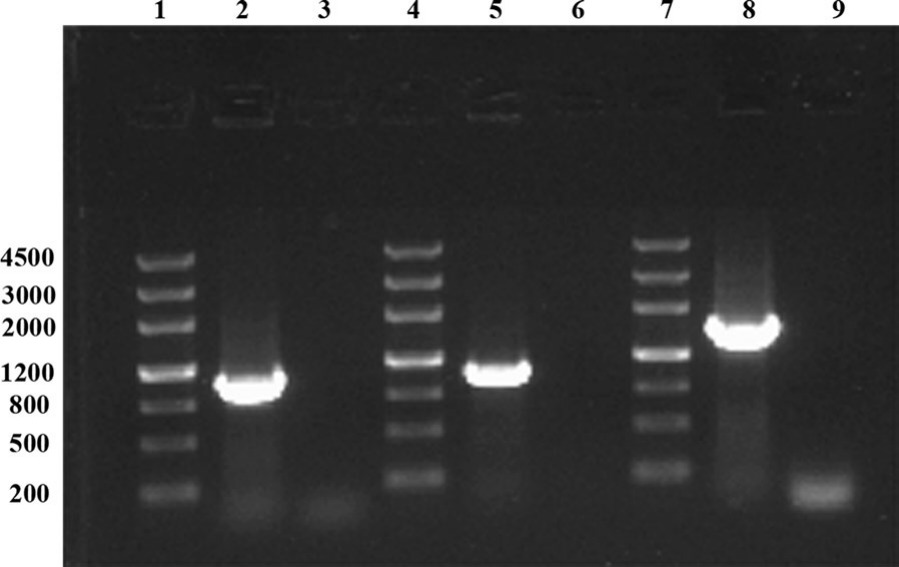
PCR amplification of three cellulase genes. *Lanes*: 1, marker; 2, cel7482; 3, control (ddH_2_O as template); 4, marker; 5, cel3623; 6, control; 7, marker; 8, cel36; 9, control

We designed three pairs of specific primers (F7482c/R7482c, F3623c/R3623c and F36c/R36c) to amplify the full-length target sequences. As a result, three specific DNA fragments with about 1.0, 1.0 and 1.5 kb were amplified by PCR from the metagenome of the anaerobic beer lees converting consortium (Fig. 2). Subsequently, the amplified fragments were sequenced, and the results showed that the nucleotide sequences of the amplified fragments were consistent with those of the assembled target sequences (id_7482, id_3623 and id_36). The nucleotide sequences of cel7482, cel3623 and cel36 have been deposited in GenBank under accession nos. KU168144, KU168145 and KU168146.

### Expression and purification of three recombinant cellulases in *E. coli*

**Fig. 3:**
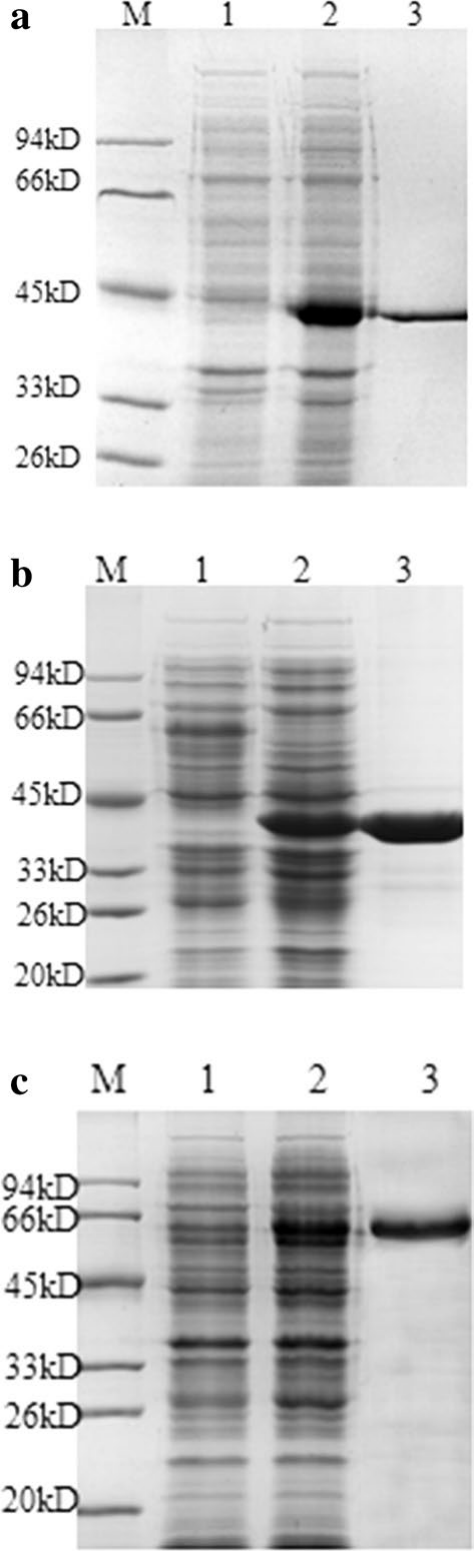
SDS-PAGE analysis for the expression of the cellulase genes in *E. coli* BL21 (DE3). *Lane 1*, total proteins from recombinant *E. coli* BL21 cells without IPTG induction; *lane 2*, total proteins from recombinant *E. coli* BL21 cells induced by IPTG for 4 h; *lane 3*, purified cellulase; M, protein molecular weight markers. **a** cel7482; **b** cel3623; **c** cel36

Expression of the recombinant cel7482, cel3623 and cel36 in *E. coli* BL21 (DE3) was demonstrated by sodium dodecyl sulfate–polyacrylamide gel electrophoresis (SDS-PAGE) analysis. After induction with 0.5 mM isopropyl-β-d-thiogalactopyranoside (IPTG) for 4 h at 30 °C, clear bands corresponding to 41, 41 and 58 kDa were observed in whole-cell lysates of *E. coli* BL21 in SDS-PAGE (Fig. 3), which matched well with the molecular masses estimated from the deduced amino acid sequences of the recombinant cel7482, cel3623 and cel36.

Three recombinant cellulases were purified from 500 ml of *E*. *coli* cultures by immobilized metal ion affinity chromatography (IMAC) using Ni Sepharose 6 Fast Flow. The purified target proteins were observed at the position of 41, 41 and 58 kDa in SDS-PAGE (Fig. 3), which were identical to the molecular masses of the recombinant cel7482, cel3623 and cel36. The data on the purification of the recombinant cel7482, cel3623 and cel36 are summarized in Table 1.

**Table 1:** Purification of the recombinant His_6_-tagged cel7482, cel3623 and cel36 from 500 ml of *E*. *coli* cultures using immobilized metal ion affinity chromatography

Purification step	Total protein (mg)	Total activity (Unit)	Specific activity (U/mg protein)	Yield (%)	Fold
Total cell lysate	328^a^, 369^b^, 358^c^	391, 321, 229	1.19, 0.87, 0.64	100, 100, 100	1, 1, 1
Ni–NTA column	11, 14, 13	316, 244, 183	28.73, 17.43, 14.08	81, 76, 80	24, 20, 22

^a^cel7482

^b^cel3623

^c^cel36

### Cellulase activity

Three purified His_6_-tagged cellulases were shown to have obvious hydrolytic activity toward carboxymethylcellulose (CMC). Cel7482 showed 1.6- and 2-fold higher specific activity than cel3623 and cel36, respectively. However, the recombinant cellulases had no activity against *p*-nitrophenylcellobioside (pNPC) and cellobiose.

**Fig. 4:**
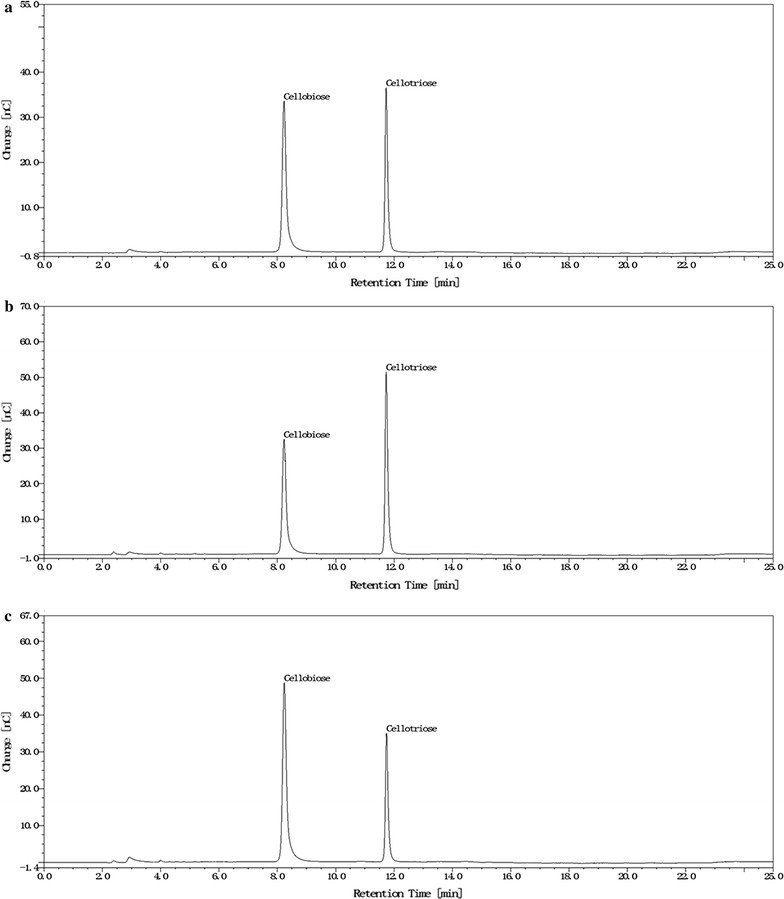
HPAEC analysis for final products of hydrolysis of CMC by the recombinant cel7482, cel3623 and cel36. HPAEC analysis was performed on a Dionex ICS5000 system equipped with a pulsed amperometric detector and a CarboPac PA200 column (Dionex). **a** cel7482; **b** cel3623; **c** cel36

To determine final products of hydrolysis of CMC by cel7482, cel3623 and cel36, HPAEC analysis was performed with enzyme assay mixtures. Cellobiose and cellotriose accumulated as products when each purified enzyme (50 μg/ml) was incubated with CMC for 1 h under optimal conditions, whereas larger cello-oligosaccharides were not visible (Fig. 4). Moreover, no glucose was detected. In contrast, no hydrolysis products were detected in reaction without enzyme.

### Enzymatic characteristics of the recombinant cel7482, cel3623 and cel36

**Fig. 5:**
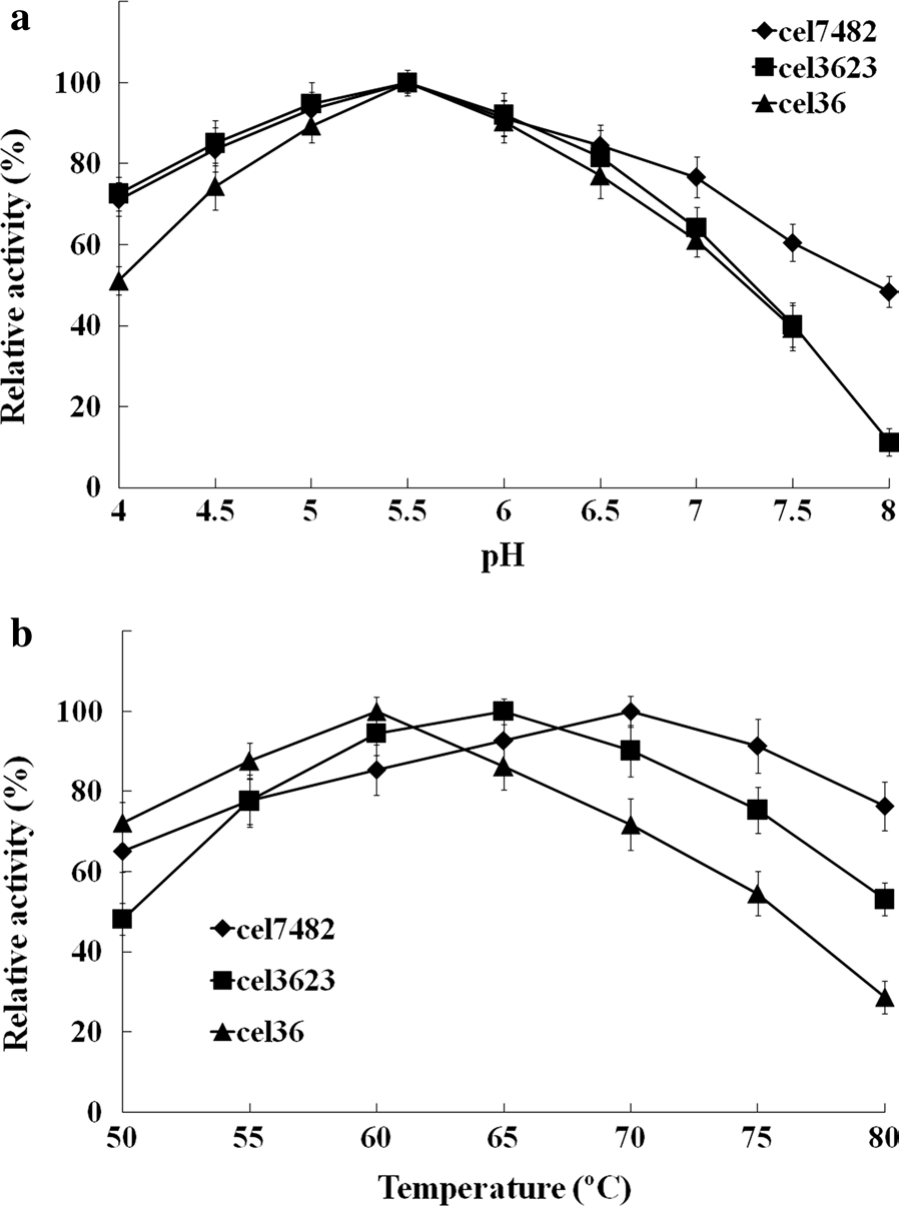
**a** The effect of pH on activity of the recombinant cel7482, cel3623 and cel36. **b** The effect of temperature on activity of the recombinant cel7482, cel3623 and cel36

**Fig. 6:**
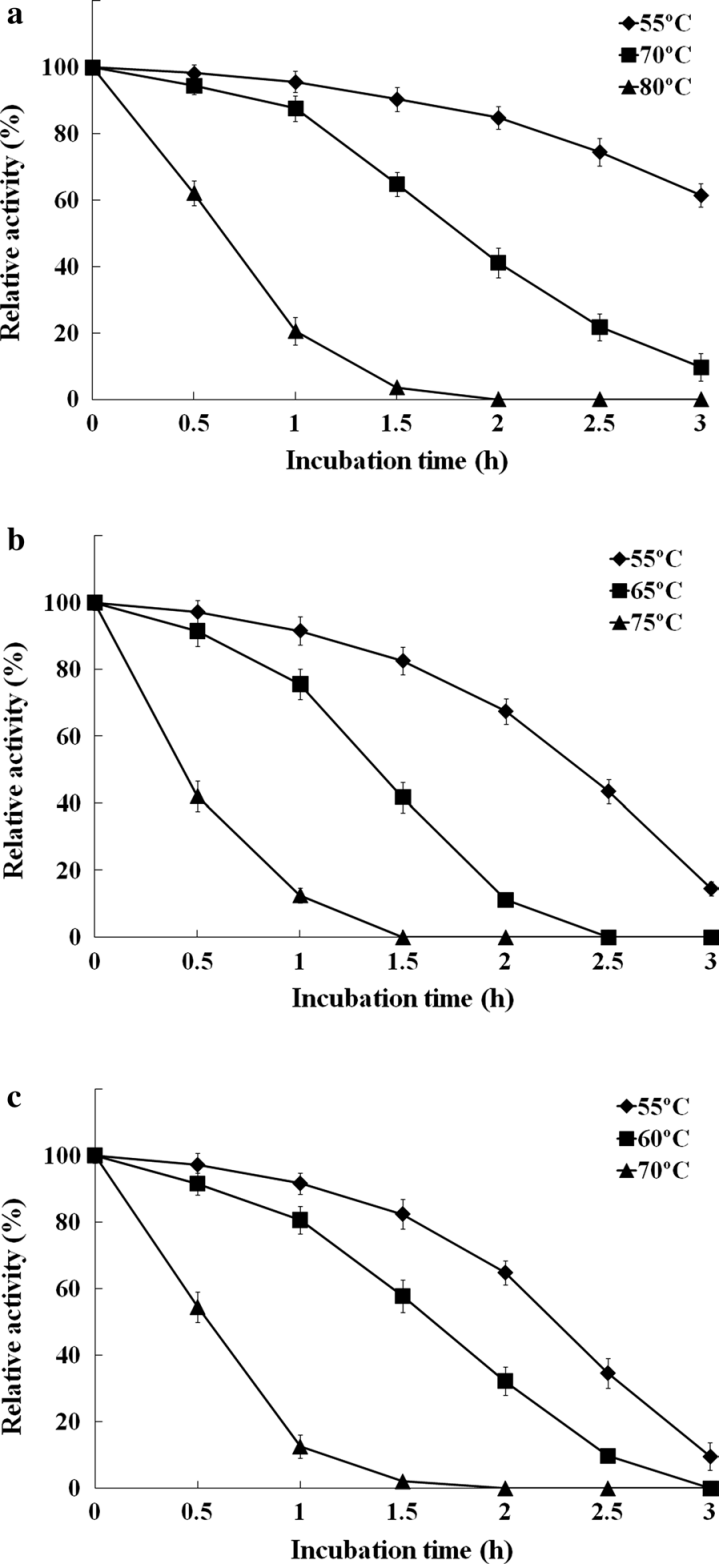
Thermostability of the recombinant cel7482, cel3623 and cel36. Each purified enzyme was incubated at different temperatures for different periods of time before determining the residual activity at optimal temperature in 50 mM citrate–phosphate buffer (pH 5.5) with CMC as substrate. **a** cel7482; **b** cel3623; **c** cel36

The recombinant cel7482 was stable over an acidic pH range of 4.5–6.5, maintaining more than 80 % of the maximum activity (Fig. 5a). The optimal temperature range for activity was 65–75 °C, within which the enzymatic activity was maintained more than 90 % of the maximum activity (Fig. 5b). The recombinant cel7482 retained about 87 % of its initial activity after 1 h of incubation at 70 °C (Fig. 6a). However, after incubation at 80 °C for 1 h, the enzymatic activity dropped to 20 % of its initial activity.

The optimal pH and temperature for activity of the recombinant cel3623 were 5.5 and 65 °C, respectively. The recombinant cel3623 retained over 80 % of the maximum activity between pH 4.5 and 6.5 (Fig. 5). After incubation at 65 °C for 1 h, the recombinant cel3623 maintained about 75 % of its initial activity. However, the enzymatic activity decreased by 88 % after incubation at 75 °C for 1 h (Fig. 6b).

The recombinant cel36 showed high activity in the pH range from 4.5–6.5. The optimal pH and temperature for the enzymatic activity were 5.5 and 60 °C, respectively (Fig. 5). After incubation at 60 °C for 1.5 h, the recombinant cel36 retained about 57 % of its initial activity. However, the recombinant cel36 was almost completely inactive after incubation at 70 °C for 1.5 h (Fig. 6c).

**Fig. 7:**
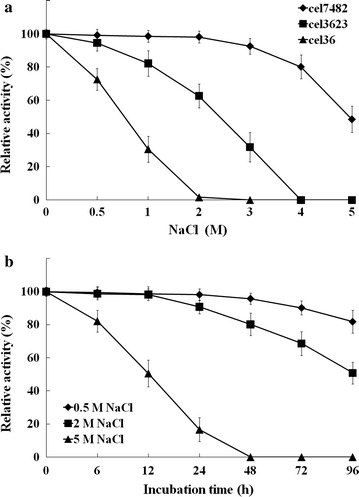
**a** The effect of NaCl on the activity of the recombinant cel7482, cel3623 and cel36. Each purified enzyme was incubated with CMC at optimal temperature for 30 min in 50 mM citrate–phosphate buffer (pH 5.5) in the presence of 0.5–5 M NaCl. **b** Halotolerance of the recombinant cel7482. After pre-incubation in 0.5, 2 and 5 M NaCl for different periods of time, the residual activity was determined at 70 °C in 50 mM citrate–phosphate buffer (pH 5.5) with CMC as substrate

The recombinant cel7482 still retained 80 % of the maximum activity in the presence of 4 M NaCl. In contrast, the recombinant cel3623 only maintained 62 % of the maximum activity in the presence of 2 M NaCl. Activity of the recombinant cel36 was almost completely inhibited by the presence of 2 M NaCl (Fig. 7a). After pre-incubation in 2 M NaCl for 96 h, the recombinant cel7482 still maintained 50 % of its initial activity (Fig. 7b).

We further tested the ability of the recombinant cel7482 to resist three ionic liquids (ILs) (i.e., [Emim]Cl, [Bmim]Cl and [Amim]Cl). The enzymatic activity remained unchanged in the presence of 20 % of one of these ILs (Additional file 1: Figure S2), suggesting that these ILs have no inhibitory effects on the enzymatic activity.

## 3D structures of cel7482, cel3623 and cel36

Sequence analysis showed that cel7482 shares 64.2 % amino acid identity with cel3623 (Additional file 1: Figure S3). From the high sequence similarity, the two proteins can be deduced to have a high structural similarity. Since there are no actual X-ray crystallographic structures of cel7482 and cel3623, we built 3D structures of cel7482 and cel3623 based on the crystal structure of a family 5 endoglucanase (PDB: 1ceo) that shares 46.0 and 47.1 % amino acid identity with cel7482 and cel3623, respectively.

**Fig. 8:**
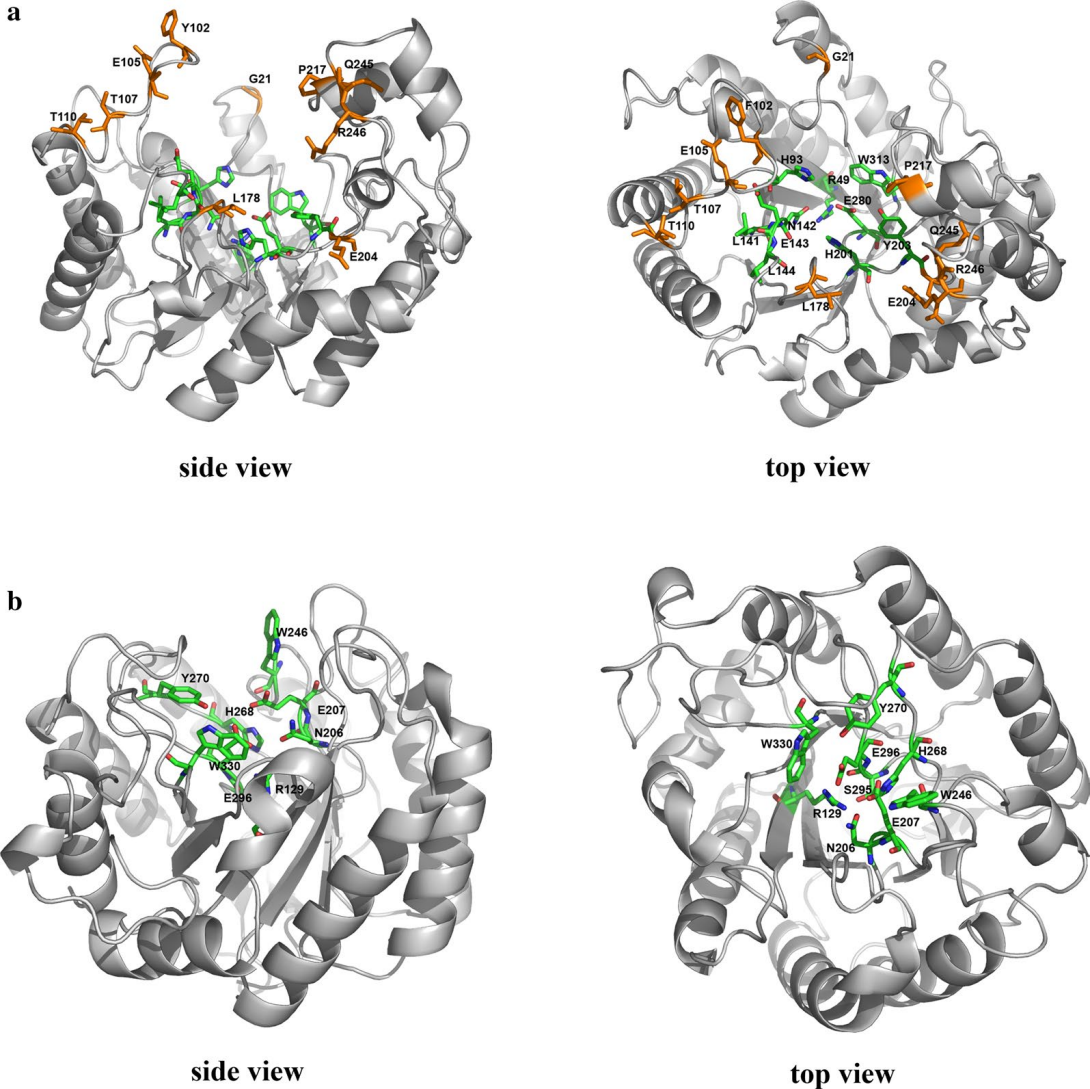
**a** The predicted 3D structure of cel7482 (*side view* and *top view*) using the crystal structure of a family 5 endoglucanase (PDB: 1ceo) as modeling template. The *colored sticks* represent the conserved residues in the active site. The *orange sticks* represent the different residues between cel7482 and cel3623. **b** The predicted 3D structure of cel36 (*side view* and *top view*) using the crystal structure of endo-1,4-β-glucanase from *Bacillus subtilis* 168 (PDB: 3pzt) as modeling template. The *colored sticks* represent the conserved residues in the active site

Figure 8 showed the predicted 3D structure of cel7482 (residues 7–340) with the active site surrounded by the inner β sheets and the outer α helixes. The predicted 3D structure of cel3623 was similar with cel7482. The important functional amino acids R49, H93, LNEL(141–144), H201, Y203, E280, and W313 in the active site of cel7482 as well as α helixes were completely conserved between cel7482, cel3623 and the template. Some of the amino acid substitutions between cel7482 and cel3623 occurred in the entryway of the active pocket of cel7482 and the substitutions contained bulky (G21W, F102S, L178F) and charged (E105S, T107K, T110E, E204L, P217K, Q245R, R246S) residues, which might affect the entry of substrate into active pocket and lead to the variation of basal and ligand-induced activities between cel7482 and cel3623.

The 3D structure of cel36 was generated based on the crystal structure of endo-1,4-*beta*-glucanase from *B. subtilis* 168 (PDB: 3pzt) that shares 33.8 % amino acid identity with cel36 (Additional file 1: Figure S4). Figure 8 showed the predicted 3D structure of cel36 (residues 76–370) with the active site surrounded by the inner β sheets and the outer α helixes. 3D structure of cel36 revealed high amino acid identity between cel36 and the template in the catalytic core. The important functional amino acids R129, N206, E207, W246, H268, Y270, S295, E296, and W330 in the active site of cel36 as well as α helixes were completely conserved between cel36 and the template.

### Secretion of cel7482 by the twin-arginine translocation (Tat) pathway of *B*. *subtilis* 168

**Fig. 9:**
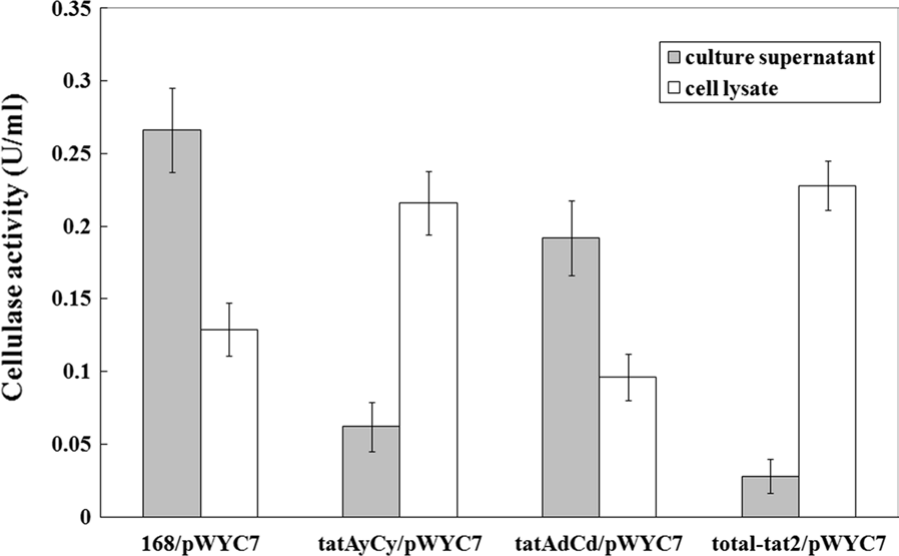
Cellulase activities of the culture supernatant and total cell lysate derived from *B. subtilis* 168 and its *tat* mutant strains. 168, *B. subtilis* 168; *tatAyCy*, *tat* mutant strain lacking functional TatAyCy translocase; *tatAdCd*, *tat* mutant strain lacking functional TatAdCd translocase; total-*tat*_*2*_, *tat* mutant strain lacking all Tat translocases. For expression of YwbN-cel7482 fusion protein, the culture of the recombinant *B. subtilis* was induction with 0.5 % xylose at 37 °C for 24 h

To secrete cel7482 into the culture medium, in this study, the twin-arginine signal peptide of YwbN (a strict Tat substrate in *B*. *subtilis*) was fused with the N-terminus of cel7482 and used to target cel7482 to the Tat pathway of *B*. *subtilis* 168. To demonstrate whether active cel7482 was secreted by the Tat pathway of *B. subtilis* 168 into the culture medium, extracellular cellulase activity was detected with *B*. *subtilis* 168 and its *tat* mutant strains. As a result, cellulase activity (0.266 U/ml) was detected in the culture medium of *B*. *subtilis* 168 expressing YwbN–cel7482 fusion protein. In contrast, cellulase activity (0.028 U/ml) was found in the culture medium of the total-*tat*_*2*_ mutant strain lacking all Tat translocases. Additionally, cellulase activity (0.062 U/ml) was detected in the culture supernatant of the *tatAyCy* mutant strain lacking functional TatAyCy translocase. In contrast, cellulase activity (0.192 U/ml) was found in the culture supernatant of the *tatAdCd* mutant strain lacking functional TatAdCd translocase (Fig. 9).

## Discussion

Complete hydrolysis of CMC by the recombinant cel7482, cel3623 and cel36 was shown to accumulate cellobiose and cellotriose as products. The recombinant cellulases could not hydrolyze pNPC and cellobiose, indicating that they lacked exoglucanase or β-glucosidase activity. Results from the NCBI BlastP search indicated that the three cellulases had highest identity with those endoglucanases deposited in GenBank. Based on product pattern and sequence alignment, the three cellulases were affiliated with endo-β-1,4-glucanases. 3D structure modeling showed that the active residues in the active site of cel7482, cel3623 and cel36 were more similar to each other compared with non-active site residues, which suggested that the three proteins should have a similar function.

In this study, the recombinant cellulases were more active in the acidic pH range (4.5–6.5). The recombinant cellulases showed high activity at high temperatures (60–70 °C) and were thermostable, which might be due to the fact that the cellulases came from a thermophilic consortium. To date, several thermostable cellulases have previously been reported [11, 12].

It is hard to obtain halotolerant cellulases by functional screening of metagenomic libraries [13]. Cel7482, which was obtained in this study by a metagenomics-oriented approach, showed significant resistance to high concentrations of NaCl. Cel7482 was extremely tolerant to 2 M NaCl and was still active in the presence of 5 M NaCl. Cel7482 may be a halotolerant cellulase due to its tolerance to high salinity environments.

Lignocellulose needs to be pretreated prior to enzymatic hydrolysis due to its inherent recalcitrance towards degradation. Although ILs have been shown to be very effective solvents for lignocellulose pretreatment, ILs strongly inhibit cellulase activity [14]. Activity of cellulases is especially influenced by the presence of chloride ions in ILs. Therefore, halophilic cellulases are regarded as promising candidates for screening IL-tolerant cellulases. In this study, a halotolerant cellulase (cel7482) was found to be resistant to three ILs (i.e., [Emim]Cl, [Bmim]Cl and [Amim]Cl), which are widely used for cellulose dissolution. Therefore, cel7482 with ILs resistance has potential for use in a one-pot process (i.e., coupling of ILs pretreatment with enzymatic hydrolysis) in which enzymatic hydrolysis is carried out in aqueous solutions of cellulose-dissolving ILs.

The Tat pathway is utilized by bacteria for the transport of folded proteins across the cytoplasmic membrane. Because *B*. *subtilis* lacks the outer membrane, proteins that are exported to the periplasm by the Tat pathway can be secreted directly into the culture medium. In *B. subtilis* 168, two active Tat translocases with different substrate preferences have previously been identified as TatAdCd and TatAyCy [15]. In this study, when fused to the YwbN signal peptide, cel7482 was secreted by the Tat pathway of *B. subtilis* 168 into the culture medium as judged from the ratio of extracellular activity (0.266 U/ml) to cell lysate activity (0.12 U/ml). In contrast, secretion of cel7482 was almost completely blocked in a total-*tat*_*2*_ mutant, indicating that the extracellular secretion of cel7482 was strictly dependent on the Tat pathway in *B. subtilis* 168. In addition, no obvious secretion of cel7482 was observed in a *tatAyCy* mutant. However, the effective transport of cel7482 still occurred in the *tatAdCd* mutant. We assumed that the TatAyCy translocase may play key roles in the export of cel7482 to the extracellular milieu. In the future, the efficiency of Tat-dependent translocation of cel7482 needs to be further improved for industrial production.

In function-based screening of cellulases from environmental metagenomes, very few active clones could be obtained from metagenomic libraries. Activity of specific cellulases may not be detected by functional screening of libraries, resulting from incorrect protein folding in *E. coli*. More importantly, the strategy cannot select new cellulase sequences prior to cloning.

Metagenomic sequencing has some advantages compared to functional screening. Diverse GH genes have been discovered by metagenomic sequencing in termite guts [16], cow rumens [17] and biogas digesters [6], and they could be utilized as a resource for screening new cellulases with industrial value. In this study, a metagenomics-guided strategy for biomining new cellulases was proposed. Cloning of cellulase genes can be performed by a simple PCR protocol without the need for construction of metagenomic libraries. New cellulase sequences obtained by metagenomic sequencing were chosen for functional expression, which significantly improved the hit rates for new cellulases.

In this work, search for conserved domains of GHs by hidden Markov models (HMMs) [18] significantly improved the accuracy of GH annotation. In total, we obtained 23 GH sequences belonging to the GH5 family by metagenomic sequencing of the thermophilic anaerobic enrichment. Among 23 GH sequences, we selected three novel GH sequences (id_7482, id_3623 and id_36) as the putative cellulases. Based on the assembled GH sequences, the three putative cellulase genes were obtained by PCR directly from the metagenome of the thermophilic anaerobic enrichment. Furthermore, functionality of the putative cellulases was verified by expression and activity assays. In the future, the rapid detection of activity of a large number of candidate cellulases can be performed using high-throughput screening systems [4, 19]. Compared to function-based screening, the metagenomics-guided strategy is particularly applicable to the high-throughput screening of new cellulases from environmental metagenomes.

## Conclusions

New cellulases with unique properties have potential for use in industrial processes. In this work, we propose a metagenomics-guided strategy to rapidly acquire novel cellulase sequences from the metagenome. When combined with high-throughput expression, the efficiency of this strategy for obtaining new cellulases may meet the increasing demand from industrial community. Cel7482 obtained in this study, which has superior enzymatic characteristics, may be a promising candidate for degradation of cellulosic biomass under harsh conditions. Secretion of active cel7482 into the culture medium simplifies the purification procedure and improves the stability of enzyme, which should lay a foundation for large-scale production of the enzyme.

## Methods

### Metagenomic sequencing and GH annotation

Anaerobic digestion sludge (ADS) collected from a local wastewater treatment plant (Shek Wu Hui Sewage Treatment Works, Hong Kong) was applied as seed sludge in this experiment. The enrichment of the beer lees fermenting consortium was carried out in a sequential batch mode in serum bottles with a working volume of 200 ml. Beer lees were applied as the primary substrate, and the temperature was controlled at 55 °C.

After 45, 75 and 120 days of enrichment, genomic DNA was extracted from 4 ml of sludge slurry from the thermophilic consortium with a FastDNA SPIN Kit for Soil (MP Biomedicals). Library size of 300 bp and reading length of 125 bp were applied for illumina high-throughput sequencing of the extracted DNA samples. The sequencing depth for the metagenomic library was 3.0 Gb.

Quality control of metagenomic raw reads derived from the illumina Hiseq 2000 platform was performed as described previously [9]. The trimmed reads were firstly assembled using MetaVelvet (version 1.1.01) [20, 21] with kmer length of 51. The assembled contigs longer than 1000 bp [22] were chosen for ORFs prediction using MetaGeneMark (version 2.8) [23] with default parameters. Next, the amino acid sequences of the predicted ORFs were screened against HMMs collected at dbCAN [24] using hmmscan [18] with E-value cut-off of 1E−4 [17] for particular GH families classified by the CAZy database [10].

### Cloning of metagenome-derived cellulase genes and their expression in *E. coli*

Three putative cellulase genes, designated as cel7482, cel3623 and cel36, were amplified by PCR from the metagenome of the anaerobic beer lees converting consortium using three pairs of specific primers (F7482c/R7482c, F3623c/R3623c and F36c/R36c). The 50-μl PCR mixture contained 2 μl of each of forward and reverse primer, 25 μl of 2 × *Ex Taq* PCR MasterMix (Takara), 1 μl of template DNA, and 20 μl of ddH_2_O. The PCR protocols were set as below: an initial denaturation at 95 °C for 5 min, followed by 30 cycles of 94 °C for 45 s, annealing at 62 °C for 45 s, and elongation at 72 °C for 90 s, with a final extension at 72 °C for 8 min. The PCR products were run on 0.7 % agarose gel, and DNA bands with the correct size were recovered using a DNA gel purification kit (Tiangen). The purified PCR products were cloned into the pMD19-T simple vector using a Takara TA cloning kit and then the ligation products were transformed into *E. coli* DH5α. The positive recombinants were identified by colony PCR and sequenced.

Three cellulase genes were re-amplified by PCR from the recombinant pMD19-T vectors using three pairs of primers (F7482e/R7482e, F3623e/R3623e and F36e/R36e), digested with Nde*I* and Xho*I* and subcloned into the similarly digested expression vector pET30a (Novagen). The recombinant plasmids, designated as pET-c7482, pET-c3623 and pET-c36, were transformed into *E. coli* BL21 (DE3). Strains, plasmids and primers used in this study are listed in Table 2.

**Table 2:** Strains, plasmids and primers used in this study

Strain, plasmid, or primer	Relevant characteristics	Source or reference
Strains		
* E*. *coli*		
DH5α	*supE44* Δ*lacU169*(φ*80 lacZ*ΔM15) *recA1 endA1 hsdR17*(r_K_^_^ m_K_^+^) *thi*-*1 gyrA relA1* F^–^ Δ(*lacZYA*-*argF*)	Takara
BL21 (DE3)	F^−^ *ompT hsdS*_B_ (r_B_^−^ m_B_^−^) *gal dcm* (DE3)	Novagen
* B. subtilis*		
168	*trpC2*	(Jongbloed et al. [15])
Total-*tat*_*2*_	*trpC2; tatAc*::Em; *tatAy*-*tatCy*::Sp; *tatAd*-*tatCd*::Km; Em^r^; Sp^r^; Km^r^	(Jongbloed et al. [15])
* tatAyCy*	*trpC2; tatAy*-*tatCy*::Sp; Sp^r^	(Jongbloed et al. [15])
* tatAdCd*	*trpC2; tatAd*-*tatCd*::Km; Km^r^	(Jongbloed et al. [15])
Plasmids		
pMD19-T	Ap^r^, TA cloning vector for PCR product sequencing	Takara
pET30a	Expression vector, T7 promoter, Km^r^	Novagen
pWH1520	*E. coli*-*B. subtilis* shuttle vector, *pBC16 ori, pBR ori,* P_*xylA*_ *, xylR,* Tc^r^, Ap^r^	Mo Bi Tec
pET-c36	pET30a derivative for expressing recombinant His_6_-tagged cel36	This study
pET-c3623	pET30a derivative for expressing recombinant His_6_-tagged cel3623	This study
pET-c7482	pET30a derivative for expressing recombinant His_6_-tagged cel7482	This study
pWYC7	pWH1520 derivative, secretion expression vector coding for YwbN-cel7482 fusion protein	This study
Primers		
F36c	5′-ATGAAAAAATACCGCTGTAA-3′	This study
R36c	5′-TCACTTTTTCTCAGCGGCCA-3′	This study
F3623c	5′-ATGACAAATTTTACAGGCTT-3′	This study
R3623c	5′-TCATATTTTTCTGCTTACTA-3′	This study
F7482c	5′-ATGAAGAAATTTGACGGTTT-3′	This study
R7482c	5′-TCACTTTCTGCTTACAATCT-3′	This study
F36e	5′-CATATGAAAAAATACCGCTGTAAAAAGAAGGGAATTCTTTGGACACTGG-3′	This study
R36e	5′-CTCGAGTCAGTGGTGGTGGTGGTGGTGCTTTTTCTCAGCGGCCAGCCATATCTCGTCCA-3′	This study
F3623e	5′-CATATGACAAATTTTACAGGCTTCAGTGCAGGAGTAAATCTTGGAGGC-3′	This study
R3623e	5′-CTCGAGTCAGTGGTGGTGGTGGTGGTGTATTTTTCTGCTTACTATCTTAATCAAATCCTCAT-3′	This study
F7482e	5′-CATATGAAGAAATTTGACGGTTTCATGGCGGGAGTAAACCTTGGG-3′	This study
R7482e	5′-CTCGAGTCAGTGGTGGTGGTGGTGGTGCTTTCTGCTTACAATCTTAACCAGTTCGTC-3′	This study

To achieve high-level expression of cellulase genes in *E. coli* BL21 (DE3), cells were grown to an OD_600_ of 0.6 and then induced with 0.5 mM IPTG for 4 h at 30 °C. To check the expression of target proteins, total proteins from IPTG-induced *E. coli* cells were subjected to 12 % SDS-PAGE analysis [25].

### Purification of three recombinant His_6_-tagged cellulases

For purification of the recombinant cellulases from *E. coli* BL21 (DE3), 10 ml preculture was inoculated into 500 ml of LB medium and grown to an OD_600_ of 0.6 at 37 °C. Subsequently, 0.5 mM IPTG was added to induce recombinant protein expression. After induction at 30 °C for 4 h, cells were harvested by centrifugation at 4 °C and 6000 rpm for 5 min. Cells were resuspended in 20 ml of 10 mM Tris–HCl (pH 8.0) and disrupted by sonication on ice (500 W for 25 min with cycles of sonication of 10 s each and 15 s pause). The crude cell extracts were centrifuged at 4 °C and 10,000 rpm for 15 min to remove cell debris and unbroken cells.

The recombinant His_6_-tagged cellulases were purified using the IMAC according to the standard procedure [25] with minor modifications. In brief, the cell-free extracts were loaded onto a Ni Sepharose 6 Fast Flow (GE Healthcare), which had been equilibrated with 100 ml of binding buffer (10 mM Tris–HCl, 0.5 M NaCl, pH 8.0). After washing with 100 ml of washing buffer (10 mM Tris–HCl, 0.5 M NaCl, pH 8.0), His_6_-tagged cellulases were eluted from the column with elution buffer (10 mM Tris–HCl, 0.5 M NaCl, 300 mM imidazole, pH 8.0). The purity of the purified cellulases was examined by 12 % SDS-PAGE. Proteins were quantified using the Bradford method [26] with bovine serum albumin (BSA) as standard.

### Cellulase activity assays

Enzymatic activity was assayed by measuring the amount of reducing sugar released from CMC using the 3,5-dinitrosalicylic acid (DNS) method [27]. The assay was performed in 50 mM citrate–phosphate buffer (pH 5.5) containing 5 μg/ml purified enzyme and 1 % CMC. After incubation for 30 min at optimal temperature, 200 μl of DNS was added to stop the reaction, followed by boiling for 5 min in water. The absorbance at 540 nm (A_540_) was measured using a microplate reader (Thermo Scientific). Specific activity is expressed as units (1 μmol of reducing sugars released per minute) per milligram protein.

The effect of pH on activity of the recombinant cellulases was studied by incubating the purified enzyme in 50 mM citrate–phosphate buffer (pH 4.0–8.0) with CMC as substrate at optimal temperature. The effect of temperature on activity of the recombinant cellulases was also investigated by incubating the purified enzyme in 50 mM citrate–phosphate buffer (pH 5.5) with CMC as substrate at different temperatures from 50 to 80 °C.

To evaluate thermostability of the recombinant cellulases, each purified enzyme was incubated at different temperatures for different periods of time, and the residual activity was determined at optimal temperature in 50 mM citrate–phosphate buffer (pH 5.5) with CMC as substrate.

To investigate the effect of NaCl on activity of the recombinant cellulases, each purified enzyme was incubated with CMC at optimal temperature for 30 min in 50 mM citrate–phosphate buffer (pH 5.5) in the presence of 0.5–5 M NaCl. Halotolerance of the recombinant cel7482 was evaluated by incubating the enzyme in 0.5, 2 and 5 M NaCl for different periods of time, and the residual activity was determined at 70 °C in 50 mM citrate–phosphate buffer (pH 5.5) with CMC as substrate.

Resistance of the recombinant cel7482 to ILs was evaluated. The purified enzyme was incubated with CMC at 37 °C for 30 min in 50 mM citrate–phosphate buffer (pH 7.0) in the presence of 20 % of [Emim]Cl, [Bmim]Cl or [Amim]Cl.

Final products of hydrolysis of CMC by the recombinant cellulases were determined by high performance anion exchange chromatography (HPAEC) on a Dionex ICS5000 system equipped with a pulsed amperometric detector and a CarboPac PA200 column (Dionex). The column was equilibrated with 100 mM NaOH and elution was performed at a column temperature of 30 °C using a linear gradient of 0.02–0.5 M NaOH at a flow rate of 0.45 ml/min in 25 min. The enzyme assay mixtures (10 μl) were withdrawn after 1 h of incubation and used for HPAEC analysis.

### Structure modeling

The peptide sequences of cel7482 and cel3623 were submitted to the automated comparative protein modeling server SwissModel [28–30] (http://swissmodel.expasy.org/) to build up the 3D structures using the crystal structure of a family 5 endoglucanase (PDB: 1ceo) [31] as modeling template. The 3D structure of cel36 was generated using the crystal structure of endo-1,4-β-glucanase from *Bacillus subtilis* 168 (PDB: 3pzt) [32] as modeling template. CLUSTALW software was used to align the peptide sequences (http://www.ebi.ac.uk/Tools/msa/clustalw2/). 3D figures were created with PyMOL (http://www.pymol.org/).

### Construction of recombinant *B. subtilis* 168 for secretory expression of cel7482

The nucleotide sequence encoding the twin-arginine signal peptide of *B*. *subtilis* YwbN [33] and codon-optimized cel7482 was chemically synthesized by BGI Inc., Beijing, China. The synthetic sequence was digested with *Kpn*I and *Sph*I and subcloned into similarly digested pWH1520, an *E*. *coli*-*B*. *subtilis* shuttle vector, to create the secretory expression vector, pWYC7. Transformation of plasmid into *B*. *subtilis* 168 was carried out using the high-osmolarity electroporation method [34]. When *B*. *subtilis* cultures reached an OD_600_ of 0.6, 0.5 % xylose was added to induce expression of YwbN-cel7482 fusion protein. After induction for 24 h at 37 °C, cells and culture supernatant were separated by centrifugation and used for cellulase activity assays.

## Additional file

10.1186/s13068-016-0557-3 The nucleotide sequences of 23 glycoside hydrolases. Three target sequences selected in this study are colored in red. **Figure S2.** Resistance of the recombinant cel7482 to high concentrations of ILs. The recombinant cel7482 was incubated with CMC at 37 °C for 30 min in 50 mM citrate–phosphate buffer (pH 7.0) supplemented with 20 % of [Emim]Cl, [Bmim]Cl or [Amim]Cl. The activity in reaction without ILs was set as 100 %. **Figure S3.** Alignment of cel7482 and cel3623 proteins. The amino acid sequences of cel7482 and cel3623 were aligned with ClustalX2.0.12. The identity or similarity of the residues is represented by (*), (:), and (.). The residues in the active site are colored in red. The different residues in the entryway of active site between cel7482 and cel3623 are colored in green and underline. **Figure S4.** Alignment of cel36 and 3PZT proteins. The amino acid sequences of cel36 and 3PZT were aligned with ClustalX2.0.12. The identity or similarity of the residues is represented by (*), (:), and (.). The residues in the active site are colored in red.

## Abbreviations

CAZy: carbohydrate active enzymes
GHs: glycoside hydrolases
SDS-PAGE: sodium dodecyl sulfate–polyacrylamide gel electrophoresis
IPTG: isopropyl-β-d-thiogalactopyranoside
IMAC: immobilized metal ion affinity chromatography
CMC: carboxymethylcellulose
pNPC: *p*-nitrophenylcellobioside
ILs: ionic liquids
Tat: twin-arginine translocation
HMMs: hidden Markov models
ADS: anaerobic digestion sludge
BSA: bovine serum albumin
DNS: 3,5-dinitrosalicylic acid
HPAEC: high performance anion exchange chromatography
