# Supplementary material for: Discovery of new cellulases from the metagenome by a metagenomics-guided strategy
Source: Biotechnol Biofuels. 2016 Jul 4;9:138. doi: 10.1186/s13068-016-0557-3 (PMC4932676; doi:10.1186/s13068-016-0557-3)
Supplement: Supplementary file 1 — 10.1186/s13068-016-0557-3 The nucleotide sequences of 23 glycoside hydrolases. Three target sequences selected in this study are colored in red. Figure S2. Resistance of the recombinant cel7482 to high concentrations of ILs. The recombinant cel7482 was incubated with CMC at 37 °C for 30 min in 50 mM citrate–phosphate buffer (pH 7.0) supplemented with 20 % of [Emim]Cl, [Bmim]Cl or [Amim]Cl. The activity in reaction without ILs was set as 100 %. Figure S3. Alignment of cel7482 and cel3623 proteins. The amino acid sequences of cel7482 and cel3623 were aligned with ClustalX2.0.12. The identity or similarity of the residues is represented by (*), (:), and (.). The residues in the active site are colored in red. The different residues in the entryway of active site between cel7482 and cel3623 are colored in green and underline. Figure S4. Alignment of cel36 and 3PZT proteins. The amino acid sequences of cel36 and 3PZT were aligned with ClustalX2.0.12. The identity or similarity of the residues is represented by (*), (:), and (.). The residues in the active site are colored in red. [file 13068_2016_557_MOESM1_ESM.doc]

**>ch4_1.gene_id_36**

ATGAAAAAATACCGCTGTAAAAAGAAGGGAATTCTTTGGACACTGGTTCTGTCTTTGATCCTGATCATGCTTTCCGGATGCGGGGAAACCCCACCAGAGGTTGTCAGTGAAACACCGCTGTCCCAGCAGGACGCCACATCCCAACAGCCACCAGAAGCTAAACCCGAAGCAACATCCGAGCCGGCTGAGCCCTCGCCGGAATCCGGTCCCACCTTTGTGGAACGCAACGGGAACCTGAGGGTGGAGGGCACCCAGCTGGTGAATGAAGCAGGCCAGCCGGTACAGCTCAAGGGAGTCAGTTCTCACGAAGTTGCTTCCTTTGGCTATCTGGTTACATACAATGCCCTGAAGCAGATGCGGGATGACTGGAATCTGACGGTGTTCCGAATAGCCATGTATACCGAGGATGCCACCGGATACATCCGAAATCCCGGTGTAAAGGATATCGTGACGCGAATTATCGATGACTGCATAGACCTGGGGATCTACGTCATCATAGACTGGCACATCCTTTATGACAATACGCCCCTGAAATACAAAGATCAGGCGGTGGAATTCTTCTCGGAAATGTCCGCCCGTTACGGGGATTATCCCAACATCATCTATGAGATCTGCAATGAACCCAATGGCGCCGACACCACCTGGGAAGGCCACATCAAGCCCTATGCGGAGGAGGTCATACCCGCCATCCGCAAGAACGATCCGGACAACATCATCATTGTGGGCACACCTACCTGGAGTCAGGATGTGGATATCGCCGCCGACGACCCGCTGCCCTATGATAACGTCATGTATGCCCTGCACTTCTACGCGGGTTCCCATGGACAGTTCCTCAGGGATAAGATCGATTACGCCCTTTCCAAAGGATTACCTATTTTTGTCAGCGAATGGGGAAGCTGTCTGAACACCGGGGATGGCCCCACTTTCCACGAGGAGTCCATGGAATGGATCAAATTCCTGGATGAGCGGAATATCAGCTATGTGAACTGGTCATTCAGCACCAAGCAGGAAGGCGCATCCATCTTGCGGAAACAGATCGATGTCAATGCCAAATGGACGGACGTCGACCTGACCGAGGGCGGATTGTTCGCCAAGTACGCCATCAAAGGCACCAAAGACACCGTATTGTTTGCCGATGGGTTTGAAACCAAGACATTCGGCCATGGCAAATGGAAAAGGAGCAATGATAACACCACCTATGAGACGGAGAATCCATACAAGGGCAACTATGCCGCATCCATCGGGAAGGACGGTTTCCTTGAAAGGGCCGTTACCACGGTGCCCTATGAAAACCTCAAACTGCATCTGGCCTATACCTTTGTGAATGGAAAACCCGGGGATGTGGTCAGAATCGAATGGTTTGACGGCGCCAATTACAACCTTGTCACCGAACTGCCGCTGGCTGAGGAGTGGACCGAGCTCGACATCGCACTTCCTGACAGCGCTTCAGGTGTACATGATTTCTCAGTCCGTATCACGGCGGCTGTCGCCGACGAGGATACCCGGCTTTTGGTGGACGAGATATGGCTGGCCGCTGAGAAAAAGTGA

>ch4_1.gene_id_1083

ATGAATATGAATGCCTTTAAAACAGGAAAATATACAAACTTATTTTTGGAATGCGGCTATTCGGAAAGTGAAATAAACGCAAGAATAAAGAAAATATGGGATACCATGTTTTTTGGCAGCGATGATGAGCGCATTTACCATGAAATAGGGGATATGGGCTACATAGTTGATACCGGAAATCTTGATGTCCGTACAGAGGGTATGTCCTACGGCATGATGATGAGCGTCCAGCTTGACAAGAAAGAAGTTTTTGACAGGCTCTGGAAATGGACAAAGACCTATATGTGGATGGATAGAGGCGACAATAAGGGATATTTTGCTTGGTCTGTTTCTCTTGACGGGGTAAAAAATTCCTACGGCCCTGCTCCTGACGGAGAAGAGTATTTTGCTATGGCGCTTTTCTTTGCCTCCGCAAGATGGGGTGACGGCGAGGGGATTTTCAATTATTCGCGTGAAGCAAGGGATATCCTTCATGAATGTGTTCACAAGGGCGAGGATGGCAGAGGATTTCCTATGTGGAATCCCCAAAATAAGCTTATAAAATTTATTCCTAACTGTGAATTTTCCGACCCATCCTATCATCTGCCGCATTTTTATGAGCTTTTTTCTCTTTTGGCGAATGAAAAAGACAGAGCTTTTTGGAAAGAAGCCGCAAAGGCAAGCAGGGAATATCTGAAAAAAGCCTGCCATCCTGTGACGGGGCTTGCGGCAGAATATGCCGAATATGACGGAACTCCAAGGATGCAGGAGGGCCATGGATTCTTTTACAGCGACGCCTATCGTGTGGCGGCAAATATAGGGCTTGACTGGGAATGGTTCGGAGCGGACGAATGGGAATGTGACTGCGCAGGCAAAATACAGGATTTTTTTGCCGCTAAAACCGGCGGTGAAGAAAATTTCATATTTAAGGTTGACGGAACAAAAATTGAGGAAAATGTTCTGCATCCGGTTGGCCTGCTTGCAGCCAATGCCCAGACATCCCTTGCTTCAAACAGCATCCATAAGATGGATTTTGTAAAAAGACTATGGGAAACCCCTTTGCGCAGCGGAGAAAGAAGATATTACGACAATTGCCTTTACCTTTTTGCCTTTTTGGCGCTTAGCGGCAATTACAGGATATATATTTAA

>ch4_1.gene_id_2504

ATGCAATGGTTTTTAGGGGGAGGATTAGTAATAATGAAAAGTAGATGTATGAAAAAGGCATTAAGCATAATTTTGGCATTAGGTATGCTATCTTCATTTTTTATGTCATCAGGTATATTCAGTGGTTTTTCACTGACAGTAAAGGCACAGGAACCGGACAATAATGATGACTGGCTCCACGTGGAAGGAAATCAGATTGTCGATATGAACGGAAATCCTGTTTGGCTGACAGGTACAAACTGGTTTGGCTTTAATACCGGAACCAATGTTTTTGACGGGGTATGGAGCTGTAATATGCGTGAAGCCCTGACAAAAATGGCAGACAGAGGTATTAATTTTCTCAGGATTCCAGTATCGACAGAGATTCTGACCGGATGGAAGAACGGAAAACCGGCTATGCCCAGAAGTTTGAATGATTACGTGAATCCGGAACTTAAAGGTCTTAACAGTCTTGAACTTTTTGATTTTGCCCTTGATGTGTGCAAAGAAGTAGGTATTAAAGTGATGGTCGATGTACATAGCCCAAAATCGGAAGCCATGGGACACAATTATCCTGTATGGTATGATGAGACTTATGACACTGAGGCATGGATATCGGCATTGGAATGGCTTACTGAAAGATATAAAAACGATGATACCATTCTTGCCATTGATCTTAAGAACGAACCTCACGGAAAGCCTTATGAGAAGCTTATGGCAAAATGGGACAATTCCACTGACATCAACAACTGGAAGTATGCCGCAGAAACTTGTGCTAAAAGAATCTTAAAAATCAATCCCAATCTGTTGATAGTTATAGAAGGGGTTGAAGTCTATCCAAAAGAAGGCTATGATTACACTGCGGTGGATGAATGGGGCAAGGAAAGCCGTTATTATTACAACTGGTGGGGTGGAAACTTAAGAGGAGTAAAGGATTATCCCATAGATTTAGGCCAATATCAAAAACAGGTGGTTTATTCACCCCATGACTACGGTCCTTTGGTACATAAACAGCCATGGTTTTATGACGGTTTTACAAAGGAAACACTTTACAATGACTGCTGGAAGGATAACTGGGCATACATTTATGAAGAGGGAATTGCACCCCTTTTAATTGGAGAATGGGGAGGATACATGGACGGAGGTCCTAATGAAAAATGGATGATAGCTTTAAGGGATTATATTGTTGAGAACAGAATTCACCATACCTTCTGGTGTTATAATGCAAACTCGGGAGATACCGGAGGACTTGTAACCCACGATTTTATAACTTGGGAGGAAGACAAATATGAGCTGTTAAAACCGGCACTATGGCAGCATAACGGAAAATTTGTAAGTTTGGACCATAAAGTGCCTTTAGGGTCTAACGGAATTTCGTTAAGCGATGTTTATGGTGATGCAATACCGACTCCAACCAGGGCTGATGTTCCCACACCTACCGTTTCTTCACCCGTAACTTTGGTTTATGGAGATTTAAATGGCGATGACGACTTCAATTCCATTGATTTTGGATTGTTGAAATTGGTCCTGCTGGGGTTAAAATCTCGGACAGAAATAAATGAGAAAGCGGCAGATGTTGATGGAAACGGTCATATAGATTCTATAGACTATGCTTTGATGAAACAGCGTTTGCTTGGTATTATTAAAAAATTTCCTGTTGAGAACTGA

**>ch4_1.gene_id_3623**

ATGACAAATTTTACAGGCTTCAGTGCAGGAGTAAATCTTGGAGGCTGGATTTCGCAGTTCTGGCAAAAAAGGAAGGAGCATTTCGACAGCTTTATCACCAAGGAGGATATCAGGCAGATAGCCGGCTGGGGTATGGATCATGTAAGATTGCCGATTGACTACAATATCCTGGAGGATGATGACAAGCCGTTTGAATACAGTGAGGAGGGCTTTTCCTATATTGACAGCTGCATAGAATGGTGCAGGGAGTATGGACTGAACATCATACTGGATCTTCATAAAGCACCCGGCTATGCCTTCCATTCGCTCGAAAGCAACAAATTGTTTGAGGATAACACACTTCAGGAGCGCTTCATCAACCTTTGGAAGACCTTTGCCAAACGTTATAGCAAATATGGCGATAATGTGGTTTTTGAGCTGCTGAATGAAATAGTCGAGCCTAATTCCGACAGATGGAATGCCCTGAGCAAAAGGGCTGTTGAGGCTATTCGTGAGCTTGACAAAAACAGGATCATCATTATCGGCGGTAATTTCTATAACAGCGTAAATACTCTGAAGGAGCTGGACCATATAGATGACGACAGGATAGTATATACCTTCCATTTTTATCTGCCCCATCTGTTCACTCATCAGGGTGCAAGCTGGGAGAAGGCAATGAGCGAGTTGAATTTTAAGATTTCATACCCTATTGGCCCTGAGGATTACAGCAAGTATATGCAGTTGTCGGAAAGCTTCAGAAGGAGCTACCAGATTAGTGAAAATATGGGCAAGGATTATCTGAGGGAGGCTCTTATGCCTGCCCTGGAATTTGCAAAGGAGCGCAAGGTAGCAGTATACTGCGGAGAATACGGTGTTATAGATACGGCAGATATGGACAGCAGGCTTCGTTGGCATGAGGATTTCTGCAGCCTTCTGATTGAATATGGCATAGGCAGGGCTGTATGGAGTTATAAGCTTATGAATTTCGCCATGGTAGATGGCAATTCAAAGGTAATCAATGAGGATTTGATTAAGATAGTAAGCAGAAAAATATGA

>ch4_1.gene_id_4475

ATGGCGAAAAGATTTTTGATTGGCGCGGCTACCGCCGCCCATCAAGTAGAGGGAAACAATACAAACAGCGATTATTGGGCTATGGAGCATATGGAATACACCAGCTTCAATGAGCCCAGCGGCGACGCCTGCGACCACTACAACCGCTATGAGCAGGACATCAAGCTGCTGGCCGAGGCCGGACTGAACGCGTATCGCTTTTCCATCGAATGGGCACGGATTGAGCCAAGACCCGGCATTTATGATGATGACGAAATTGAGCATTACCGCAAGGTGCTGGAGTGCTGCCGCAAAAACGGCGTCGAACCCATTGTCACCATGCATCACTTTACAAGCCCCAAGTGGCTGATCGAGCAAGGCGGTTGGGAAAACGAAACCACAATTGAAAGATTTGCCGATTACTGCAAATATGTTGTGGAGCGTCTTGGCGATCTGTTCACCTACGTCTGCACTATTAATGAAGCCAACATAGGCCTGCAAATTGCGGCCATTTCCAAACGATACATGCTGATGATGCAAAAAAATCAAAATGCCGGAGGCAATGTGCAGGTCGGCATCAATCTGGAAAATCCCATGCCGGAGCGCACTAAAAAGCAGACAGAGGAAAATATGCAGGTTTTCGGAACCCCTCAGCCCCAAGTCTTTGTATCTTCCCGCACGCCAAATGGCGATCTTCTGGTCATGCGTGCCCATCAGTCGGCCAAGGCTGCCATGAAATCAGTCAAGCCGGAGCTTCAAATCGGCATTACCCTTTCTCTGCATGATATTCAGGCGCAACCTGGCGGAGAAGCCGAAGCAGAACGCGAATGGGACAAGGAATTTTTGCACTACCTGCCATATATTCAAGATGACGATTTCTTTGGCCTGCAAAATTATACCCGTATGGTAATAGGGCCAGAAGGAATCCTGCCTATACCTGACGGAGCAGAAACTACACAGATGGGCTATGAATTTTATCCTCAATCCCTTGCCAACGTAATCCGCACAGTACATAAGCGCCTTCCAATCCCCATTATGGTGACAGAAAACGGCGTAGCGACAAGCGACGATCATCGCCGCAAAGCATTTATAGGTGAAGCTCTTGAAGGGTTAAAGGATTGCCTTAAAGACGGGATTCCAGTAATCGGCTACTGCCACTGGAGTTTACTTGACAACTTTGAATGGCAAAAGGGATATTCAATGACTTTCGGACTTGTTGCAGTTGACAGAAAAACTAAAGAAAGACGTCCGAAACCAAGCCTTACATATCTCGGACAATTTGTTAAGGAGATTGTACAATGA

>ch4_1.gene_id_6210

ATGTTTGCTGGAAAAAGAAATCTTAAAAGAGTTATTACATTGTTTTCTTGTGTCGCGATGCTATCAACAACGATATTGATACCAATGAAGAACGTTTCAGCAGCAGGATATGATTACTCATTGGCCCTAAAAAATGCCATCGGATTCTATGATGCGAATAAATGCGGCAAAGATGTAAAAACAAACAATATTTTTGACTGGAGAGGTCCGTGTCATACAAAAGACGGACAGGATGTAGGCTTAGATTTAACAGGAGGGTACCATGATGCCGGAGACCATGTAAAGTTCGGATTGCCTCAAGGATATACAGCATCAGTGCTTGGGTGGGCATTGTATGAATATGATGAAGTGTTTAAAGCAACCGGCAACAAGACGAAAATGCTTGAACAGCTAAAGTACTTTACAGATTATTTTTTAAAGAGCCATCCCGATGCCGATACTTTTTACTATCAAGTAGGAGACGGTGAGGAAGACCACAATTATTGGGGACCACCGGAACTTCAATCGGAAGACAGGCCTACTAAATGTGTGGCAAATAAAAATAATCCTGCCAGTGATGTTTTAGGAGAAACGGCAGCTGCTCTTGCACTTATGTATCTGAATTATAGGGATATTGACTCTACATATGCCAATAAATGTCTTCAGGCAGCAAAAGAGTTATATAAAATGGGAACAACCAATAAAGGTCCTGGAGATGGGCAGTATTTTTACAGATCCACCAGTATTTATGATGACTTGGCTTGGGGAGCGGTATGGTTGTATACTGCAACCGGAGACAGCTCATACATTAATGATGCAAAAGAATTTATTGTTGTAAAAAATGAAAGTGGAGATGATCCTTTTAAGAAAAGATGGACAATGTGTTGGGATGACATGTATGTTCCAGCCTTAGTTAAATTGGCTGAGATAACCGGGGAACAGATTTATAAAGATGGTGTAGAATATAACCTAAATTATTGGATGAATGATATTCAAACAACTCCAGGCGGTTTGAAATACTTGAATTATTGGGGAGTATTAAGATACGCAGCAGCTGCTTCAATGGTTGCAGCAATATATTATAAACAGAATCCCAACAAAGGTTATCTTGACTTGTTGAAATCTCAGATTGATTATATTTTAGGTGACAATCCTGAAAAAATGTCCTATGTGATAGGAATGGGCAACAAATGGACCCAACATCCTCATCATAGAGCTGCACAAGGGGCAATCGGTTATGCAGACAATGCCAATACAGCACCGGCGAAGTACCTTTTACTCGGTGCTTTGATAGGTGGTCCCGGTCCCGATGATGTATTTAAAGAAAGTGTATATGAATATCAATATACTGAAGTTGCCATTGACTACAATGCAGGTTTCGTTGGGGCACTGGCAGCTACAGTTAAACATTTTGGAAATTTAAATATTCCCGATGTGACAAAAACCCCGATTCCAAATCCGACACCAACTCCTACAACCAATAAATATCTTGTCGGAGACGTGAACCATGACAATAATATTGATTCCATAGATTATGCGCTGATGAAAAGCTATTTGCTCGGTATGAAACTTCCGGAAAATACGTTCTTTAAGAATGAAGCCGATGTTAATGGTGATGGGGATATAAATTCTGTGGACTATGCACTTTTGAAACAAAGGCTTTTAGGTATGATTTCTAAATTCCCTGTTGAAGAATAA

>ch4_1.gene_id_6480

ATGAGTTTTTTAAAAGTAGATGGTCCTTATATTAAGGACGAGACCGGTAAGAAGATATTCCTGAGGGGAGTATGTTTTGGCGGATGGTTAAATATGGAGAACTTTATCACCGGATATCCTGGTGCAGAAAGTTCTATTAGACAGTCTATAAAGGAAGAGCTTGGGGAAGAAAGATATCAGGTATTCTTCTCTAGCCTGTTAGACTCATTTATAACTGAGGGTGACTTTAAATTTCTTAAAGAGATAGGAGCCACCGCAGTAAGAATTCCATTTAACTACAGGCATTTCGAAGATGATATGAATCCAGGCAATTACAGTTCTTCTGGATTTTACTACCTTGACCGGGCTATTGAGTGGGCAAAAAAGTATAACATATATATCATCCTTGACCTTCATGCTGCACCAGGATGGCAAAACCAGGGGTGGCACTCTGATAATCCTTACGGAATATCACTGTTCTGGAATGATAGACATTATCAGGAAAGGGTTAAGGGGCTATGGGTTTATATAGCAGAGCATTACAGGAATGAGCCACAGGTTGCCGGATATAATCTTCTCAATGAACCTAATGCTCCTAATATAGATATTTTAAAGAGGATATATAGAGAATGGATAGAAGCAATAAGAAATACGGATAGGAAACATATAATATTTATTGAGGGTAATCGATACTCTCAGATCTTCGAGGGATTAGACGAACCGTTTGACGATAATTTAGTATACAGTTCTCACAATTATACAATAGCAACTCACAGGGCAAGAAAATATCCAGGTTATGTTGGGGATGTATATACAGATAAGAGTTGGATGGAAAGGATATTCCTGGAGAGGAATAGATGGATTTTAGATCATAAAAAACCGTCCTGGGTAGGCGAGTTTGGAGCGTTATTTGATGGAACCATTGATTCTCCAACAAATGCAGATAAAGCCAGACTTTTAGCATTAAAAGACCAGCTGGAGATATTTAATAAGTATGAACAACACTGGACAATCTGGACTTATAAGGATGTGGGGGTTCAAGGATTAGTAGTTCCTAAGGAAGACAGTGAATATATGAGAAGGATCAGACCTGTCTCTGAACTGAAGAAGAAGTTAGGGCTTGACCCCTGGACATCAAGAGGTTATGGATTACTGAGGGGCGAAACTGCAAGGATGGTAGAGACCATAGCTCAGGCGGTATCTGAAATTGTAAAAGACTATAGTCTTGATTATACCTCACTTACCAGGGAATTGGGGGATAGATTACTGAGTGGTGCTATTAGTAGCTTCCTTGCACCACTATATGCATTTCAGTTTATGGATATGACTGAAGAAGAGATAGAAAAGATGCATCATATGGCATTTGAGTTTTCTAACTGTGAGAAGAGACTCTACCTGATAGAAGTGTTAGAGAGTGCTTTAAAAAATTAA

>ch4_1.gene_id_7082

ATGCCGAACTTGTTTTTAGGTTCGGCATTTTTTGCATTGTTGTTTGAATACGATTACAATGAAACGGAGGCTCCCAAAATGAAAAAAAGCTTTGTTTTGTCCGCTGTTATCCTTTCCTTTATTTTAGGCGGCTGCTCAAATTCCGCAAAATCCTCTGACATTACAGTGCAAACCGAAACTGCTCAAACTTCAAGCGAAACATCATCCGCGTCCTCGCAAGAAAATGCAACCATTGTCCTTTCAATCCCGGAAATAAATCTGCAAAATAAAACCCTGCCCGAAAATGAGGCAATTGATTTTGTACGCAACATGAAGGCCGGATGGAACCTCGGCAATACCTTTGACGCAGTTTCCGACGGAGGATACAATGAGGCAACGGAAATGAAAATCGAATCTTCATGGTGCGGAGTTGCCACTACAAAAGAAATGATAGACGAAATTAAAAAAGCAGGATTTAATACCGTGCGCATACCCGTTTCATGGCACAATCACCTTACCGACGGCAATTTTACAATAAGCCAAAAATGGCTTGACAGGGTAAAAGAAGTCGTTGATTATGCAATAAGCAACGATATGTATGCAATAATCAACATTCATCACGACATAGACAAGGCATACTATTATCCATCAAGCGAATGCCTTGAAAATTCCAAGAGATATATTTCTTCAATATGGACCCAGCTTTGCCAAAAATTTGGAGATTATGACAACCGTCTTATTTTTGAATCGGTAAACGAACCGCGCCTTGCCGGCACAAACTACGAATGGTGGTTTGACAAAAACAGTGCACAGTGCATTGACGCGGCAAACTGCATAAACGAACTTAACCAGCTTTTTGTGGACACGGTACGCGCATCCGGCGGAAACAATGCCTCGCGCTATTTGATGTGCCCCGGATATTCGGCATCGGCAAGCTCGGCGCTTATGGACGAATTCAAGCTTCCAAAAGACAGCGTGCAAAACAGGCTCATAGTTTCAGTGCATGCCTATACGCCGTATAATTTTGCCCTGCAAGGCCCTAACGAAAGCGGCAGCAAATCCGATTTCAAAGCCTCCAATTCTTCCGATACAGGTGAAATAACAAGCTTTATGGACAGTCTTTACCAAAAATTTATAAGCAATGGAATCCCTGTCGTAATAGGAGAATTCGGGGCAAGGGACAAAAATTCAAACACTCAGGCAAGAATTGATTTCTCCGCATATTATGTTGCATGCGCCCGCGCAAACGGGATTACCTGCTGCTGGTGGGACAACAATGCTTTTTCCGGCAGTGGCGAAAACTTCGGAATTTTTGACAGAAGAACACTTACATGGAAATACCCCGAAATAAAGGATGCAATAATAAAATACTGCCAGTAA

>ch4_1.gene_id_7419

ATGACCATGAGAACTCATATATGGCTGTTGGTATTTTTGATAATTGCTATAGCAGGAGGGATTATCGTGTTCGACAATCTTGACAATAATAATGATACCAGCGTCATCAACGACAATAAAAAGTCTGAAGAATTAAGTGTATTCGACTACAATAAGAAAATTGGTCATGGAGTCAACATAGGTAATGCACTTGAAGCACCAGTTGAGGGCTCTTGGGGTGTTTACATATCGGATGATTATTTCCCTATCATCAAAGAACGTGGTTTTGATTCTGTACGTATTCCCATTCGCTGGTCTGCTCATATTGAAGAAAATTATCCATACAAGATAGATAAAGAATTTCTTAATAGGGTAAAACACGTTGTTGACGAGGCCTTGGAGAATAACTTGATTGTGATCATAAACACACACCACTTCGAAGAAATGTATCAATCACCGCAAGATCACAAAGAAGAGTTGATAGAGATTTGGAGACAGATTGCCGATGCGTTCAAAGACTACTCTGATTCTCTTTACTTTGAGATATTCAACGAACCTGCGCAGAATTTGACTTCTGAGCTCTGGAACGAAATCTATCCTGAAGTCTTGGACGTGATACGTAAGACAAATCCGAACAGAGTGGTCATCATTGATGTTCCAAATTGGTCGAATTACTCAGCTATCGATGAGTTGAAACTTGTAAGAGATGAACACATCATCGTCTCCTTCCATTACTACGAACCATTCACATTTACACACCAAGGCGCAGAATGGGTTAATCCACAATTACCTGTGGGTGTAGAATGGAGAGGTAGTGATTTCGAAGTTGAGCAAATCAATGTGCATTTCAAACATGTCAGTGACTGGGCGAAGAAGAATAATGTACCCATCTTTCTTGGTGAGTTTGGTGCTTATTCAAAGGCTGATATGAAATCTCGTGTCTTGTGGACAGAAGCAGTTAGGAAGACGGCTGAGAAATTCGGTTTTTCGACTGCATACTGGGAATTTTGCTCTGGATTCGGCCTTTACGATAGCTCAACCTCACAATGGAATGAACCTCTAACTAGTGCAGCACTTGGAAAGTGA

>ch4_1.gene_id_7421

ATGTTTCCAAAAGACTTCGTTTTTGGTGCTTCGATGTCAGGATTTCAGTTTGAAATGGGTAATCCTTTATCAAAGGAAGAGATAGACCCAAATTCCGATTGGTTTGTCTGGGTAAGAGAAAGTGAGAATCTTGTAAACGGAATTGTGAGTGGTGATATGCCAGAAAATGGTGCATGGTATTGGAAACAGTATGATAAAGTTCATTCACTGGCAGTTGATTTTGGTATGGACACACTGAGGATAGGCATAGAGTGGTCGAGGATCTTTCCAAATTCCACCAAGCATATACCGTTCGATGCCCCTAATTTGCTTGAAGAACTTGATAAGATAGCGGACAAGTCCGCAGTGGTACATTATAGAAAGATAATAGAAGATATGAAGAGTAAGGGATTGAAAGTCTTCGCCAATCTCTATCATTTCTCATTACCACTTTGGCTTCACGACCCAATAGCCGTGCATAAAGGAAAGCAGACGGATAAATTAGGTTGGATAAGTGATGATACACCGATAGAATTTGCAAAATTTGCAGAATATATGGCTTGGAAGTATTGTGATATAGTAGATATATGGGCATCGATGAATGAGCCACATGTTGTAAGTCAGTTAGGGTATTTTATCACAAGTGCTGGATTTCCACCTGCATACTTTGATCCAGAATGGTACATGAAGAGTTTGAAAAACGAGGCAACTGCGCACAACTTGGCTTACGATGCCATTAAGAAGCACACAACAAATCCTGTTGGAATCATATATGCATTTGCTTGGGTTGATACCGTTTCTCCAAACGACAGTGACATATTTGAAGAGGCTATGGAAATGTCCAATTGGCGATTTATGGATATGATAAAAGACAAAATAGACTACATAGGCGTTAATTATTACAGCAGAATGATGATAGATAGGTTACCTACGGTGGTCAAACTCGGGCCACTTGAAATGAAGTGGAACTCACTGAGAGGTTATGGTCAATCTTGCGTTGAAGGTGGAGTTGCGCTTTCTGGTAGGCCCGCAAGTGATTCTGGTTGGGAAATCTATCCAGAAGGGTTGTATAAATTGTTGAAAGCGGTATCACAGCGATACAAAGGTATACCGTTGTTGGTGACGGAGAATGGTATATCTGATGAAAAGGACAAGTACAGACCATTTTACATAATTTCTCATCTTTATGCAGTTGAAAGAGCAATCGAAGATGGTGTTGACGTGCGAGGTTACCTGCATTGGTCGATCATCGACAACTACGAATGGCCCAAAGGATATACGAAACGTTTTGGACTTGCATATACAGATTTTGAGAAAAAGACATATGTGCCAAGGCCTTCGATGTACATCTTCAAGGAAATATCGGAGAAAAGGACGACAAAACACTTGATAGGATACGACCCATACAACTTGATCAATTTTTGA

>ch4_1.gene_id_7983

ATGAGAATTAAGGCAATAACATTTTTTGCAGTCATTGTATTATTTCTTGGCGGACTTTTTATAGGTTCGGACAAGGTTTATGCGTCACAAAGGAAGCGGCCACATCTTAACAAGGAAAGGACAACATTTGTAGGGGATAACGAGCAACCTCTCAGGGGGCCATATACATCTACGGAATGGACTCCGGCCGTTCCATATGAACAGATTGAGGGCATTAAGAAGCTGGGCTTTAATGCTGTCCATCTGTATGCCGAGTGCTTTGATATAAATTATCCCAATCCGGGAAGTACGGCGCCCGGTTATGCAGTAAATGAGGTTGACAAGATTGTTGAGGCCACAAGAGAGCTTGGGCTTTATCTGGTAATCACCATAGGAAACGGCGCAAATAACGGCAACTATAACCTGAAATATGCTGAGGATTTTTGGGCTATATATGCCAAACGTTATGCAGATGAAAGCCATGTCCTCTTTGAAATACATAATGAGCCGGTTGCATGGGGGCCACCGTATTCATCCCAATATGCCACTCCGACAGGAGCGGTGGACATGCAGATATCTGTTTATAAAATTATCCGTAAATATGCACCCGATACCCCGGTATTGCTTTTCTCGTATGCTGTACCATCAGGTAAATCAGGAGCAAATGATGCCATGAAGGATATACGCATTTTCAACAAGGCTGTTTTTGGGGATGAAAATGCGGTCTGGACCAATGAGGCGGTGGCCTTCCATGGTTATTCCGGCTGGAAATTGGCTTCTGAGTTCGTTGCAAGCATGATCAGCGAGGGCTATCCCTGCTTTATGACGGAATTTGCAGGAGGCAGTTGGGGCAGCGGCAAGGGCGGACTGGATGCCGAGATGGCATATCAGTTGGAGCACCTTGGAATTTCCTGGTTAACCTTTCAGTATATTCCGCCAACAGGGGTATCGGATAATGTGGCCATACCGGAGCATTTTTCGGCTATTGTAGAAAATACCGGACTATCATGGAAGCCGGATTATGGCGATTGGCCTGTCCTAAGAGGTATATATGGCAATGATGGCCTTCCCAGAAAAACAAAGGAATTCAGAGTTGGGGACAGCATGACCGGAACCACACATATTGAAGCTGAGGATTTTGACTGGGGTGCCGACGGCATATCCTATCATGACAGCACTGCAAAAAATCTGGCAGGAAAATACCGGCCAGATGAAGCTGTTGATATAGAGGAGTGCGGCGATGACGGCGGCGGATTTAATATAACACACGTAAAAGCTGGAGAATGGCTGGAATACACCATATGGGTGCAAAATCCCGGATATTTTGACATATCCCTCCGTGTAGCCTGCCAAAAGCCCTCCTGCGTGCAGATTAGCTCATCGGGTCAGGACAAGAGCGGACGCTGGGAGTTGGAAAGTACGGGTGGCGACGGAATATGGGCAACACAAAGCAGGCCTGTATATTTGGATTATGGTATCCAGAGGCTGAGAATAACCTTTTTGTCCGATGATATAAAGCTAAACTGGTTTGAACTGGCTCCCGTTAAGGAAGGGCCCATCCCGGACGGAAGCTATAAATTGCTTAACAGAGCAACCGGTCTTGCTATGACGAAGGAAGCGGACAAGGATATCGTTACCCTATCGGGCTATAATGGAGCAAATGAGCAAAACTGGTTTATTATGCATACCGGTGGTGGCCACTATAAGCTTAGTTCAAAGGGTAAATGGTGGAGCATGAGCAACAGGGTCATAATTGTACCGGAAGCTAATGGCTATTACCGTCTTATCGATGTGGAAAAGGGCATGAGTCTGCAGGCGACAAAAGAAGGAGATGGCTTTGTATTGGGCAGCGCTCCTTACAGTGGAGCAGACAGCCAGCAATGGGCAATATCTGATACAGATGCACCGGCAATTCCCAGCGGATTAACTGCTATCCTTGAATTATCGGGACAGGCAGGTAGCATAAGCTGGGACGGTATGCCAGGGGCATTAAGCTATAAAATCAAGCGTTCAACAAGCAGCGGCGGGCCTTATAAAACAATAGCAAGCGGTGTTGAAAAGACAAGCTACATTGATACCACCATAAGATCAGGCGGCAAATATTACTATGTAATCAGTGCGGTTACAAAAGAAGGTGAAAGCCTAATTAGTGCTGAAAGCAGACTGCGTTTTCCTGAGCTTACAGGCAGCATAATAGGTACCGATGGCTCATGGTCCAATTCAGGAAATACCAAGCATAAGGTATTTGATAAGGATATAAACTCCTTCTTTGATTCGCCAATCGGTAATGGCAGTTGGGCAGGACTTGATTTTGGCAGTGAGACTAAATATGTAATAACCCAGATTAGCTATTGTCCGAGAGCAGATTTCCCGGGACGGATGGTTGGAGGTGTATTTCAGGGGGCAAATCAGGAAGACTTCAGCGATGCGGTTACTCTGTATACAATAACAGAGCAGCCGGCAGCGGCTAGATTTACTACTGTGGAAATAGACAATAAGGAAGCCTTCAGATATTTCCGTTATCTTTCACCAAATGACGGATATGGCAATGTTGCCGAGATCGAGGTTTTTGGTTATGCACAGACCGGTGCCCAAGCCGGCCGGCAGTCGGATATGGCAGGGGATATTTTCTCGGATGGGCAAATTGATACAATTGACTTAATAGTACTTAAGAAGCACCTTATGGGAATAGAACAGATTCAAAATCAAAAGCCTGCCGATTTAAATTCCGACGGCTGTATAGATGCTCTTGACTATGCGATAATGAAACGGTATCTGGCTGGAGCAATAGCAAGTCTTCCTGAGTTGGCTGAAAGAAAGATAAGCGCTTTGTCGGTATTGTCAGCAAGCAATAGAAAACCAAATTTGAAATCTGAAAGCTCTTGTGGCTACGGTGACTGCTTCGACCCTTTACTGTCAGTCGGGAAACTAACAGCAATTGCAATTTTACCTGACAGGAAAAAGCAGGGTCTGATTTTATAA

>ch4_1.gene_id_8091

ATGAAAAAAAGGCTTTTAGTTTCTTTTTTGGTGTTAAGCATAATTGTAGGATTACTTTCTTTTCAGTCGCTTGGTAATTACAACAGTGGTTTAAAAATCGGTGCTTGGGTGGGAACCCAGCCGTCAGAATCAGCAATTAAGAGTTTTCAGGAACTTCAGGGTAGAAAGCTTGATATTGTCCACCAGTTTATTAACTGGTCAACTGATTTTTCCTGGGTAAGACCTTATGCCGACGCTGTTTATAATAACGGCTCAATATTAATGATTACCTGGGAACCTTGGGAATACAACACTGTAGATATCAAAAACGGTAAAGCGGATGCTTACATAACCAGAATGGCGCAAGATATGAAAGCCTATGGCAAGGAAATTTGGTTAAGACCTCTTCATGAAGCCAACGGAGACTGGTATCCATGGGCCATAGGATATTCTTCAAGAGTAAACACAAACGAAACTTACATAGCCGCTTTCAGACATATTGTCGATATTTTCCGTGCCAACGGAGCCACCAACGTCAAATGGGTGTTTAATGTAAACTGCGACAATGTAGGTAACGGCACAAGTTATCTGGGTCATTATCCCGGAGATAATTATGTAGACTACACCTCAATTGACGGATACAACTGGGGTACCACTCAAAGCTGGGGAAGCCAATGGCAAAGCTTTGATCAGGTTTTCTCCAGAGCCTACCAAGCTTTGGCATCAATAAACAAACCCATCATTATAGCAGAGTTTGCATCAGCTGAAATAGGCGGAAACAAGGCAAGATGGATTACAGAAGCATATAACTCTATAAGAACATCCTACAACAAGGTAATTGCTGCAGTATGGTTTCACGAGAACAAAGAAACCGACTGGAGAATCAACTCAAGTCCTGAAGCCCTTGCAGCATACAGGGAGGCAATAGGAGCCGGTTCATCAAATCCTACCCCTACTCCAACTTGGACCTCTGCTCCACCATCAAGCTCACCAAAGGCTGTCGACCCCTTTGAAATGGTTAGAAAAATGGGTATGGGAACAAACCTCGGAAACACTCTCGAAGCTCCCTATGAAGGCTCCTGGTCCAAGTCTGCCATGGAATATTATTTTGATGATTTTAAAGCTGCAGGATATAAAAACGTAAGAATCCCTGTAAGATGGGACAACCATACAATGAGGACATACCCGTATACCATTGACAAAGCCTTTTTGGACAGGGTTGAGCAAGTGGTTGACTGGTCACTTTCAAGAGGTTTTGTTACAATTATAAATTCTCACCATGATGACTGGATCAAGGAAGACTATAACGGAAACATAGAACGGTTTGAAAAGATATGGGAACAGATTGCGGAAAGGTTTAAAAACAAATCCGAAAATCTTCTGTTTGAAATCATGAATGAGCCTTTCGGTAACATTACAGACGAACAAATAGACGACATGAACAGCAGAATATTAAAAATAATCAGAAAGACCAATCCAACCCGTATTGTTATAATAGGCGGAGGTTATTGGAACAGTTATAATACGCTTGTAAACATTAAAATTCCTGATGACCCATACTTAATCGGAACTTTCCATTACTATGACCCATATGAATTTACTCACAAGTGGAGAGGTACATGGGGTACTCAGGAAGACATGGATACTGTAGTAAGAGTATTTGATTTTGTTAAGAGTTGGTCTGACAGAAACAATATCCCGGTATATCTTGGAGAATTTGCCGTAATGGCTTATGCCGACAGAACTTCCCGTGTAAAATGGTATGATTTTATAAGTGATGCGGCCCTGGAGCGCGGTTTTGCATGTTCCGTATGGGATAACGGCGTTTTTGGTTCATTGGATAATGACATGGCTATTTACAACAGAGATACCCGTACCTTTGACACTGAAATCCTCAATGCACTATTTAATCCCGGAACATATCCGTCTTATTCTCCGAAACCTTCACCAACTCCAAGACCGACCAAACCGCCCGTAACACCGGCTGTCGGTGAAAAAATGCTGGATGATTTTGAGGGTGTGTTAAATTGGGGTTCATACTCCGGTGAAGGTGCAAAAGTTTCAACAAAAATTGTGTCCGGAAAAACAGGAAACGGCATGGAAGTCAGCTACACCGGGACAACGGACGGCTACTGGGGAACAGTATACAGTTTACCGGACGGCGATTGGTCAAAATGGCTTAAAATCTCTTTTGACATTAAGTCCGTTGACGGTTCTGCCAATGAAATCAGATTTATGATTGCTGAAAAAAGCATAAACGGTGTGGGAGACGGAGAACACTGGGTTTACTCAATAACTCCCGACAGTTCGTGGAAAACTATAGAAATACCGTTCTCCAGCTTTAGAAGAAGACTTGATTATCAGCCGCCTGGACAGGATATGAGCGGTACTTTGGATCTTGACAATATAGATTCAATTCACTTCATGTATGCCAACAACAAGTCGGGAAAATTTGTCGTAGACAATATCAAGCTGATTGGTGCTACTTCCGATCCGACTCCTTCAATAAAACACGGAGATTTGAACTTCGATAATGCAGTGAATTCTACAGACTTGTTAATGCTTAAAAGGTATATCCTCAAATCTTTGGAACTCGGTACATCTGAGCAGGAGGAAAAATTCAAAAAAGCGGCAGATTTAAACAGGGACAACAAGGTCGACTCCACTGACTTGACAATTTTGAAAAGATACTTGCTGAAAGCCATCAGTGAAATACCCATATAA

>ch4_1.gene_id_8092

ATGAAATGCAGATATATGGATTATTTAATAAACTGTGAGTTGGACTCTTTGCCTAATGAAAAATCCGAAAAAATAAACAAACATATAAGCACCTGCATGGACTGCAGAAATTATCTGGGTGCACTTATGATATCCAAAAAATATATTGCCAAAGAACCGGAAATGGATAAATATTTTTACATGCGGGTTATCAATGCCATAGACCCTGACAGATACAAGAAATCGAAACTGACTTTTAAGATTCTGTCTTCATTGGAAAGGCTAAAGCCTGCTTTTAAAGCGTCTTTGGGAACTTTGGCAGTCTTTGTGGCAGTAGCTTTGTTAATAACCGGCGGAATTTTTGACAATCTCGGCAACTGGATTGCCAAAAGCAGCAATAACCGCTCCAATACCGGTGAAACAACAAACCTGACCTTTTTGCGTACAGACGGTCAGAATATTGTTACCGGTACCGGTGAAATCTTCCACATAAGAGGTGTTACCCTTACAAACAACTTTTGGGGCAACTGGGTCAACGGAGAATCGGAAAAATTGCAAAGTCAAGGTATGGACCCTATTATACGCCCTCTTGTACAGGATGCCTGGGTGCTCACTGATGATGATTTTGAGCGTATTAAAGACCTGGGCTGCAACACGGTTTTATATGATATCAATTACCAGCTTTTTGCAGAAGACAATCCAAACAGGGAAGAAAATCTCAAAAAGCTCAAAGAACATATAAGGCGTTTTTCCTCAATGGACATATATACGGCTGTTATGCTAATGGCTCCTCCGGGACTGGATTCGATCAATGACGCCTACGAAAAGTACAAACACGGCTCAGAACGTATAAAATCTGTGTTTGAGGATGATACCTACTACGAACAGTGGGTTGAGATGTGGAAGTATTTGGCCGAAGAACTTAAAGACTTTAAAGGTGTGGCAGGATATGGACTTATAAACCAGCCAAGAGCCCCGAGTGAAAGTGAAGGTGGAATCGGGATATTCAGGGAACGCCTGAACAATGTATGCAGAGAAATACGTAAAATTGACAAAAATCATATCATATTTGTTCCCGAATATAACAGCAGAGAGGCCAATCCCGGCGAATCCTACTGGAACGAAAAAACAAATAGTTATGTAATAGACAACGGTGAGCAAGGTATTATCTGGGAAAGAGGTTTGGTAAAAGTTGATTCATCAAACGTAGTATACTTGTTCCACTTTTTCGAACCATACAACTTTGTCAATGACGGTGTCGGAGATTTTGATGCCGAAAGCCTTGAAGCTCAAGTCAGAAAACGTTATGAATGGGCTAAAAATGTCGGCAGGGCTCCGCTTCTTACCGAATACGGAATCTCCCGGGTAAACAGCGTAGACAAACGTGTACAATGGCTTGAAACCGTTCACGACATCTTTGATAAATACGGTATCTCGGCTTCATACTTCCAATATAAAAATGCCGTAGGTGCTTTTATAAATGTGAAAACCGGTTTTAACGCTTTATACGGAGAATATGTCAGCTGGGATAGTGAAATCGGCCTGAATCCCTTTTACTTTGTAAATGAACACGTTGCCACATCCGCAAAAGAAAATCATTTTGATGAAGCACTTAAAGAGTATTACCTTAAAGGTAAAAACCTGAAAAAAATTTCAATACTGGACAATCAGCCCATTCTTGAAACATTGCAAAATTTTTGGAAATAG

>ch4_1.gene_id_8270

ATGAAAACTATAAAGGTTGGACTCTTGCTTGGACTGGTGCTGCTTTTGGGATTAGTCTTGCCGGGATGTAATAAGGCAAAGGATAACGAACCAAAGGATGCTGCTGTTGAGCTTTCACCAACAGTTGCTCCGGATGCACAGGAAGCAAAAGGCTCAGGTGATGAGTCCACGCAGCATGATGAAAAGGAGATGGCTGAAGCTGATATGAAGGAAGAGCTTATAATACATAAACCAACTGCAGGAGATATTGTTAACGATATTATTATAGGATGGAATCTTGGAAATTCCCTGGATAGCTACAGCGGTAATGTCACAGGGCTTGATACGGAGACCTGCTGGGGAAATCCCAGGATAACAAAGGAATTAATTGATATGGTAAAGGAGGCCGGCTTTAATGCAGTCCGTGTACCGGTCACCTGGTATAACCATATGGATGGGAAGCATAAAATTGATGATGCATGGATGAACCGGGTGGAGGAAGTAGTAAATTATGTACTTGATAACAATATGTACTGCATTATAAATGTACATCATGATACAGGAGAAAAAGGTTGGTTGAGAGCCAGCAGTAAAAATCTTGAGCAGAACAAGGAAAAATTTAAGGCCATTTGGGAACAGATAGCGGACAGATTTGGCGATTATCCGGACAAATTGCTGTTTGAAAGCTTTAATGAAATTCTTGATGATAAAAACAACTGGACAAATCCCGCTGCTGAGGCTCTTGATATTACAAATGAGCTGAATCAGCTTTTTGTAGACACAGTACGCGCTTCCGGCAAAAAAAACGATACCAGATGCCTTATTGTAAATACCTATTGCGCGGGCGCAGGAAGGGATATTGTTAAAGGCTTTACGCTTCCGAAGGATACGGTAAAGGACAAGCTGATTGTATCAGCGCACATTTACCAGCCCTATCATTTTACCTCGGAAACCTCACCGATAACCACCACATGGGGTGTCGGCAAAACAACTCTGGAATCCTATATTAAAAATATGTATACCTACTTTGTACAAAAGGGGATTCCGGTTATTATCGGTGAATTTGGGGCAGTTGACAAGAATAACAGGAATGAGCGCCAGTCATGGCTAAAATACTATGTAGATACCTGCAGCAACTATGGAATTAAATGCTTCTGGTGGGATAACGGTAATGAATATAAAATCTTTAACAGAAAAACTATGCAGATAGCAGAACAGGAGCTTATAGATATCATGCTTACTGAAGCAAAAGGCGGCGATTATGTGCTGGATCGGACCTTGTATGGAGATGCTGACGGAAATGGAATTGTAAATGATGAGGATCTGCTTTTGCTTCGGAAGTATCTGAAGGGAGAAATTGATAAGGTAGATAATTGTGATATGAATAAGGATGGAGTTATAGATGCTCTGGATGAGGAGCTTCTTGCAAATCAGCTTGAGCTGGCTGCCAATATGTGCGCCAATCCGAATAACTGGACCAGCTGGGTAAATACCGGCAATGGTGCCGCCGCTCAGATGAGCCATCTAGATAATGGAGTACAGATTGCAGCAACAAATAGCGGGAAAAATTCGTGGGATGTGCAGATGTCCTATACTAAGCTTATCCTTGAAGAGGGGGCAAGCTACAAAATTTCCTTCAACTACAGCGGAAGCCCCGCCCAAAGCATGCCCTTCCATATAATGCAGGATTATGGCGAGTACAAAACATACTTTAGCCATACATTGGAATATAAGGAAGAGCTGCAGCGTTATGAGGGAGTGTTTACCATGACGAAGGCTACGGACAGAAATGCCCGGATTACATTTGACTGCGGTGCAAGCAAGCTTGATGTCCCCTATACTGTTACAATTGAAAATCTGGTGATTATTAAGCTCAAGTAG

>ch4_3.gene_id_1882

ATGGAGGATATTATGCGATTTAAAAAATGTTTTATATTATTGGCCACATTGGTCCTGGTCATGCTTCTTGCGGCAGGTTGTTCAGGCGAGAAAGAGGCTAAGGAAGGCGGCAGGGAAGAAAATGAGCCTGTAACCGAAGAGTCCGGTACCAGAGACAGCAAAACCGATGGTGACGAAGAGGGCGGCCCAAGCGGCCGGGAGACCGGTGATACAAACAATCCGGACGGCGAAGCGGGCGATGGTACCGGACGGCCTGCTCCGGTTGAAAACCATTATTGGCCGGAACCTGGTACAATGAGGGATATGACATCGTATGAGGTCGTACTGGATATGGGCCTTGGCTGGAACCTGGGCAATACCTTCGATGCCTGCGGCGACTGGATCTCCGGTGGAGTAAGCGCCCATGAAACCGCATGGGGAAGCCCGATAACCGCAAGATACATGTTTGAAAAGCTGAAGGAAATGGGCTTCCGCAGTGTCAGGATTCCGGTGGCCTGGTCAAACATGATGCAGGAGAACTATACCATATCCGTCCTTTGGATGGACCGCGTCGAAGAAGTGGTTAATTATGCCCTGGACAGCGGGCTGTATGTAGTTTTGAATATCCACTGGGACGGCGGCTGGATTCTTAACTTCTCAACCGAATATGACGAAACGATGAAGAAGTATACAAGGATATGGGAGCAGATTTGTGAGCGCTTCAGAAACTACCCCGACTACCTGATATTCGAATCCATGAACGAGGAAGGGCATTTCGACGATATCTGGAACCGCTACGCGTGGAACTACAAGGAAGAAGACAAAATCAGGGCATATGACATATTAAACAGCGTTAACCAGAAGTTTGTTGATATAGTCCGCGGTTCCGGCGGAAACAACGAATTTCGTCATTTGCTGATTGCCGGATATGCAACGGATATTGACCTGACCATCGATCCGGCGTTTAAGATGCCCGAGGATCCGGCCGGCAGGCTGATGATATCGGTTCACTACTACACCCCGTCAACCTTCACAATACTTGAGGAGGATGCCGATTGGGGCAAGTCCGAATATACATGGGGAACCGATGAAGATGATTATCAGAGAATCAGGGACGACTTTGAGAAAATGAAGGTACATTTCATGGACAAGGGCTATCCGGTCATTATAGGGGAGTATGGCGCGACAAAGACAAACAAGGACCCGGACAGCGTAAGGAACTACATTACGGCGGTGGCAAGGACGGCGTACGAGATGGGAATGTGCCCGATGCTTTGGGATGCCAATGGCCATTACAACAGGCTATTGGGAACCTTCAGGGATGAGCAGCTTCTTAAGAACCTGCAGGAAATTATGGCAATGGAAAGGTAA

>ch4_3.gene_id_4516

ATGAAAATAATTTATAATGTAAAACGAAGAAGAAAAATCTTCATATTGGGCTTATTCGGCCTGGTGGCTGTTTCAATAATTGTTTCAGCAATGTTCATCATGAAAAACGATGGAGACAGTAAGCAATCCGGTTCAATATCCGTTCCCAATAAAACAAAACCTTCTACAGAATTTTCAACCGTGTATTCCGATAGATTTATCAAGCTTTTTGGAGATATTTATACTAAAGGTTATCTAAGTGAAGAGGGTATTCCTTACCATTCAATTGAAACCCTTATTGTAGATTCACTGGATTACGGCCATCTTACAACAAGTGAAGCTTTCAGTTATATGGTTTGGCTTGGAGCTACTTACGGTAAGCTTACCGGTGACTGGTCATATTTTATAGATGCGTGGGATAAAACAGAGCAATATATTATTCCCGACCCTCAAAAGGATCAACCCGGTATCGAGGCTTATTCACCGAAAATACCTTCACAATATGCACCGGAAGCCAATTCTATCAGTGGCTATCCCGTTGCTGTCAGTGAAAGTGCACCCACGGGCATTGATCCCATCTCCGATCATCTGGCTTCTGCTTACAGCTCAAAAACTTTATACCAGATGCACTGGCTTCTTGATGTGGATAACTGGTATGGATTCGGAAATCATGGCGACGGTACAAGCCGTTACTCGTATATAAATACATACCGAAGGGGACCTGAGGAGTCTGTATGGGAAACCATTCCGCATCCCGCCTGGGAAGACTTTAAATGGGGTGATGTCAACAAGAGTGGTTTTCTGTCTTTATTCAGCAGTTCTACTCAACCTGCAAAGCAATGGCGTTACACCTCATCACCTGACGCCGATGCCAGACAAATACAGGCAACTTATTGGGCATACCTCTGGTCTAAAGAACAAGGAGTAAATAAAGAACTTAAACCCTACTTTGATAAAGCTGCAAAAATGGGGGATTATCTCAGATATTCTTTATTTGATAAATATTTCAGACCCATAGGAGTTCAAAACGGGTCAAATTTCGGCAAAGGCTATGATAGTTGCCATTATCTTCTTTCCTGGAATATTTCATGGGGAGGAGATATAGGCGGAACTTCAAGTTGGAGAAACGGAAACTCCCATCTGCACCAGGGATATCAAAACCCGGTTGCTGCCTTTGCTTTATCCAGTGAATCGATACTTAAACCAAAATCAAAAAATTCGAAAAAAGACTGGGAAAAGAGCCTAGAGCGGCAGCTGGAATTATTTCAGTACCTTCAAAGTTCTGAAGGTGCCATAGCCGGTGGTGTAACCAATAGCTGGAACGGGAAATATGAAAAGTATCCTGAAAGAACAAGTACATTCTATGATATGGCATACGACTGGCAGCCTGTTTATCATGATCCGCCCAGCAACAATTGGTTTGGATTTCAGGCAAGGTCAATGGAAAGAATCATGGAATATTATTATCTGACCGGAGATAAGAAAATAAGTGAGCTTTGCAGGAATTGGGCATCATGGGCAATGGAAAACACTTATCTGAACAATGACGGCACATATCAAATTCCTTGTACTTTAGAATGGAGCGGTCAGCCTGATACATGGACAGGAAAACCTTCTTCCAATAAGAACCTTCATTGCACAATTAAGGACTGGACAAACGATGCGGGTGTTGCTGCTGCCTATGCAAAAGCACTCATATATTATGCTGCCGCCACCGAAAAGCATGAAAAGGCTCTCAATGATCAGGCCAGAGAAACTGCAAAACAGCTTCTTGATAGAATGTGGAAAAATTACAAGGATGATTTGGGTATTTCCACTCCCGAATCAAGAGACGATTACAAACGCTTTTTTGATGAAGTGTATATTCCTTCCGACTTTTCCGGTACTAATGCTCAGGGAGCGGAAATAAAAAACGGAATAACCTTTATTGAAATACGTCCCAATTATAAAAATGATCCCGGCTATTTAGAGTTGGAAAAAACCGTTAACAGTGGCAATGCTCCTCAAATGAAATATCACCGTTTTTGGACCCAGGCTGAAGTTGCAATGGCAAATGCAATGTATCACATTTATTTTGAACGGAAGGGAAAGGTAACGGTACCCGGTATTAAACCGGAAGGAAATAAAGGTGTTTCTCAAACATCCAGTCCGGATAATACACCCGTTATTTCTCCTACTTCAACTTTAGAACCGACACCTACCGTTACACCTACCCAAACACCGGAGCCTACCAATACACCGACAATATCGCCTACAGGTACTGATAATTCTACGCATACACCGACAATTAAACCTACTCCGACCCAAAAAGCAACAAATACACCTACTATTAAGCCTACCAATACTCCAACTAAAAAGCCGACTAATACTCCCACTAAGAGACCTACCAACACTCCGACTAAGAAACCGACTAATACTCCCACTAAGAGACCTACCAATACTCCGACTAGAAAACCTACTAATACGCCTACGCCGACTAGAAGACCTACCAATACTCCAACCAGAAGACCTACTAATACGCCGACTAAAAAGCCGGATAATGGGCCAGTAAAACTTTCGCTGCAGTATACTAACAACAATTTTAATGAAACAACAACCAATGTTAATGTAAGTTTAAAAATTACCAATAGATCCGATTCCAGCGTTAAGCTTTCGGATGTGAAAATAAGATATTACTATACTGTTGACGGCGATCAATCACAGAATTTCGCTTGTGACTGGAGTAACGCCGGTTCTTCCAATGTCACCGGCCGGTTTGTAAAACTTCCTTCTCCAAGGAGCAATGCCGATTACTATCTTGAGATAGGATTCACAGACGGAGCCGGAAGTTTAGATCCGAACGCCACCGTAGAAATTCAGGCAAGATTCTGGAAATCGGATTGGTCAAACTATCATCTTAATAATGATTACTCTACCGGTTCCGGAAGAATAGCTCTCTACGTTTCAGGCAATCTGGTTTCCGGAAGTGAACCATAA

>ch4_3.gene_id_4577

ATGGCGATACTGCTGTTATTGTCAATGGTTTTATCCTTAATGGCATGCAAAAAGAAGGGGGATACTCCTTCGGAGCCTGAGGGGCAGCCTACTGAACAGACTGAAACCGGGGATACGGGAAAAGAAGATGTTGTAACAAAGGAAGCATTTCTTGACGACGGAAGGTTAACCCTGTCGGAGCTGTCATCCGTATCCTTGTCCGAGTTGACTGCGGTCGAAATGGCCCGCGTGATGGGGATCGGGATTAACCTTTCAAATACCATGGAAGCCTGCAATACGAACAACAGAGTTCCAAACCTAAGTCCGACTTATTATGAGCAATTGTGGGGACAACCGGTGACCACGCAGGAGATAATAAGCGGCATGAGGGAGGCGGGCTTTAAGTCCCTTCGTATTCCCGTGGCATGGATGAATGCCATGGATTTCGAAAACGGCGATTATACATTGGGAGAAGCCTATCTTGACCGCGTGGAGGAAATAGTCAATTACGCATTGAACGCGGATATGTTTGTTATGATAAATGACCACTGGGATCATGGCTGGTGGAGCATGTTCGGCCATCCGGACCAGGAGGTCCGCGACAAGGCCATGGATATGTACATTGCCATGTGGACCCAGATTGCCGAAAAATTCAAGGATTACGATTACCGCCTTATATTTGAATCAGCAAACGAAGAGCTTGGCGACCGTCTGAACGACCGGACCGAATTCAGTCCTACCGGAGGTACCCTTACGGAAGACGAGCGGTATGAAATGGTCAGGAAGATCAACCAGACCTTCGTCGATGTGGTGCGCAGGACAGGTGGATACAACGCCGACCGTTTCCTGCTGGTAAAGGGATATAATACGAATATTGACAAGACTGTTGATGATCGATTTGTAATGCCCACCGATACTGCTAAAAACAGGCTCTTTGTCGGTGTACACTACTACGAGCCCTGGGGCTACTGCGGAGATACCGCAGGAGTAAGCGCATGGGGAACCTTGGCCGAGGTTGAGAAAATGCACAGCACCCTTGCCAAAATGAAGAAGTTCACGGACCAGGGATACGGAGTCATTATCGGAGAGTGGGGTGTCCTTGATAACAAGGGTGAGGACAGGTACAACTATTATTTCAATTTCCTGAATTTGAACGAAAAATACGGTTATGTACCTTTCCTGTGGGATACAGGAGGCATATACAGCAGAAGGGAATACAAGATCCGTACGGACACCGATGACGAGGCAATCAGTAAGATCATCAGTCTTTTAAAGAATTTTGACGTTAAGACACTGGCGGATATGACCGTTGAAGAAATAGTCGCTAATGCCGAGGATAACCTGGCACTGGCTATAAAAAAGGCGGAAAGCAGGCCCCAGTTTACATATGGTGATGACGAGGCATACGCATGGATTATGCTGACCAGCGGTGACTGGGCGATACAATACAGTGTCGGTGACGTATATGAACCCGGTTCGAAGCCGGATGGGCTGGTTGCCACGGATGTAAAGATTGAAGGACCCGGCACCTACACGGTTGCACTGGATATTACGGGCACAGACCAGGGATACGCCAACGGTCTCAAGTTTTCTGCGGTTGGTATAGTCAATGCCGAGAAATTATGGCCCGGATATATAATAGAGATAAAGGAAATCCTTATAAACGGCGAACCGGCTGAACTATCCGGAAAGCCGTATACAACCAGTGACGACGGCAATACAACCAGGGTAAACCTTTATAACGAATGGGTAGGCAGCCTGCCTCCCGAGGCCAGGACACTGGACGGAGATTTATCAGATGCGACACCGGTACCCCTTGAGAAATATACAGGAACAAAGATACAGACCATACAGGTTACATTTGATTATATCGAGGGGTAG

>ch4_3.gene_id_6812

ATGAAACCGCTTGAAGGCTACTTGCACGGCGCAAATCTCGGACACTGGCTGTCGCAGTACGGGCACAGGGGTTCGGAGGAGTACTGGAGCACCTACATAACGAAAAAAGACATCGCCCGTATGGCGGAGTGGGGGATAGACCATGTCCGGCTGCCGGTAGACTACATGTTTTTTGAGACGGACGATGAAAAGCCGATGGTCTACGACGAATCCCGCCTTGCGTATATCGACAATACCATCGAATGGTGCGGCGAGTATGGGATTAACCTCATTTTGGACCTGCACCATGCGCCGGGGTTTGTCTTTTTGGGAGGCGAAAAGAACGACCTTTTCACAAACGAGAAAAACCGCGCGCGGTTCCTCGCGATTTGGCGCATGTTCGCCAAGCGTTACAGCGGCATAGGCGACAGGCTTATTTTCGAACTTTTAAATGAGCTTGTCTGGGAAAACAGCGACCCGTGGAACGAGCTGTGGCCGGAGGCTTGCGAGGTAATATGGAAGGAGAGCCCGCACAGGAGGATAATAGTCGGCGGCAACCACTTCAACAGCGCAAGCGAGCTGAAAAACCTTAAGGTCACGCCGGACGAGCGCATAATCTATAACTTCCACTTCTATGAGCCGATTTGGTTTACGCATCAGAGCGCGCCGTGGGTTGATTTCTTGCGCGACTACAAGCGCCAGATAGAGTACCCGTTCCGCCCCGCGGATCACGCCGAGTTCCGCGACGGCGCAATTGTCCGCGCGTACAGCGGGTACGAGGTAGTTGACAAAAAGTATCTCGAGGACAGGCTTAAACCGGTGGATAAATTCATAGAGGAAACCGGCAAGACCGTCTACTGCGGCGAGTACGGCGTGTTTTTCGTTGCCCCGCGCGAAAGCGCAATCCGTTGGCTTAACGATGTCAGCACACTCCTAAAGGAGCGCGGCATCGGACGGGCCGTTTGGAGCTACCGCGGGTTTGCCACTATCACGTCGCCGGACAACAAGACGTGGGACGAAGAGATGGTAAAGGCAATCGTGATGTAA

**>ch4_3.gene_id_7482**

ATGAAGAAATTTGACGGTTTCATGGCGGGAGTAAACCTTGGGGGATGGATATCACAATACGGCAAGGCTGGAAAGGAACGCTTCGATACCTTCATTACAGAAACAGATATCAGGCAGATTGCAGGCTGGGGAATGGATCATGTAAGGCTCCCCATCGATTACATGGTGCTTGAAGATGATGACAGGCCATTTGAGTACAAGGAAGAAGGCTTTTATTATGTGGACAGCTGCATCAGGTGGTGCGAAAAACATAACCTGAATATCATACTGGACATTCACAGAGCGCCGGGGTATGCATTTTACTTTTTAAATGAGAATACATTGTTCACGGATGAGCACATGCAGAAAAGGTATATCGGGCTGTGGCGATGCATGGCCGAAAGGTATAAGGGCTATGGAAACAACCTGGTCTTTGAGCTGTTAAACGAGCTGGTCGAACCAAACAGCAGCAGATGGAACAATCTCAGCAAAAGGACGGTTGAAGAAATACGCAAGATTGACAGGAACCGCAAAATAATAATCGGCGGCAACCTCTACAACAGCGTCAACACCCTGCATGAGCTTGACCGGATCGATGACGACAACCTGGTTTACACATTCCATTTTTATGAGCCGCATATATTTACCCATCAAAAGGCCGGTTGGGAGCCGCTGCTTAAGGATTTGGAAGTCGACGTAACATATCCTTCGACGGAGGAAACGTATGAGGTTTATTATCCTGAAAGATTAAGGCAGCGGTATTTATTCGGCAAGGTGGTCGACAAGGAATACCTCAGGAGATATATGCAGCCCGCCCTTGATTTTGCAAGGGAGAGGAATGTGCCTCTTTACTGCGGAGAATACGGTGTAATAGACCGGGCTCCCATGGACAGCAGGTTAAATTGGCATAGGGATTTGAGCGATCTGCTTATAGAATATGGGATAGGCAGGGCGGTGTGGACTTATAAGCTTATGAGCTTCCCGCTGGTGGATATTAATTCGGCCGTAATAAGCGACGAACTGGTTAAGATTGTAAGCAGAAAGTGA

>ch4_3.gene_id_8646

ATGGGGGGAAAGAACTTTATGAAAAAAGGAAAAAAAATATGCTTACTTGTTGCTGTGCTAATGGTCATCTCAATGATTTGTTTTTCATCACGCTCTGAGCAGCCGATGGCCGTTACTGTTGACAGCAACAATGACGACTGGTTGCATTGTAAGGGCAACAAAATTTACGATATGTACGGAAACGAGGTATGGCTGACCGGTGCCAACTGGTTTGGTTTTAACTGCAGTGAAAACTGTTTCCATGGTGCTTGGTATGATGTTAAAGGCATCCTTTCCGACATAGCTGACAGAGGTATTGGATTTTTAAGAATACCCATCTCCACCGAGCTGCTTTACAGCTGGATGATTGGCAAACCGAATCCGGTATCCAGTGTAACAGCCAGCAATAACCCGCCTTATCATGTTGTAAATCCTGATTTTTATGACCCTGAAACCGACGATGTAAAAAACAGTATGGAGATATTTGACATAATAATGGGTTACTGTAAAGAACTTGGAATTAAGGTAATGGTTGATATACATAGTCCTGATGCCAACAATTCCGGACACAACTATGAATTGTGGTACGGCAAGGCAGGAAGCACATGCGGCGTTGTTACTACAGAAATGTGGATAGACACCTTGGTTTGGCTGGCCGACAAATACAAAAACGATGACACTATCTTGGCTCTGGATTTAAAAAACGAACCCCACGGTAAGAGGGGATATATGGCAGAAGTTCCCGAATTGCTTGCAAAATGGGATAACTCCACTGACGAAAACAACTGGAAATACGCTGCTGAAAGATGTGCAAAGGCAATTCTTGATGTTAACCCAAATCTGTTGATAATGATTGAGGGTGTAGAACAATATCCAAAGACCGAAAAAGGTTATACCTATGAAACCCCCGATATCTGGGGAGCAACCGGTGATGCATCTCCATGGTACGGTGCATGGTGGGGCGGTAATTTAAGAGGAGTTAAGGATTATCCTATTGATTTAGGACCTTTAAACAGTCAGATTGTATATTCTCCCCACGATTACGGTCCTTCAGTTTATGCACAGACATGGTTTGACAAAGATTTTACAACTCAAACCTTATTGGACGATTATTGGTACGATACCTGGGCTTATATCAATGACCAGGGAATTGCCCCCCTTCTTATCGGTGAGTGGGGAGGACATATGGATGGCGGTAAAAACCAGAAATGGATGACACTTTTAAGAGATTACATGATTGAAAACCGCATACACCACACATTCTGGTGTATTAATCCGAATTCGGGAGATACCGGTGGATTAGTAGGCAACGACTGGAAAACTTGGGACGAGGAAAAATACGGATTATTGAAACCTGCATTATGGCAATCCGGTGGCAAATTTATAGGACTTGATCATCAGATACCTCTTGGTAAAAACGGAATGTCTTTGGGTGAATATTACGGTATGCCTACACATACAACAAAACCCACATCGACGCAAAATGCGACACCGACTCCTACAAAGGCAGCAAACACTCCGACTCCGACTTCAACAAACGGTTCGATTCTCTATGGCGATGTGAATGATGATGGATCAGTAGATTCATTGGACGTTACAATTTTGAAGAGAATTGTTCTGAGAAAATATAATGGTTCATACAATAAAGAAGCAGCAGATGTAAATGCTGATGGTGCTATTGATTCATTAGACGTTTCAATCTTGAAGAGATTCGTTCTCAGAAAAATCGATAAATTACCGTATTAA

>seed.gene_id_3350

ATGTGGAATATTTGTACTGCTTTACGCCGAATATTATACAGCACCGTACTGATCAGCGGAATTCTTTTCGCCGGCTTGTTACAAGCTCAGGGATTTTTGCATCAAAACGGGAAAATGATTCTCGATGGCAATGGGAACGAAATGATTCTCAGAGGTATAGGAACCGGGAATTGGTTGCTTCAGGAAGGTTATATGATGAAAACTGCCGATATCGCCGGCACACAAACCCAGTTTCGCAATAAACTCATTGCTACCATAGGCGTTGAAAAAACGAATATTTTCTATCAGCATTGGCTCGACAACCATTTCACCCGGCGCGATGTGGATTCGATGAAGGTGTGGGGATTCAACAGCGTCCGTGTGGCCATGCATTACAAGTGGTTTACTCTACCCATTGAAGATGAACCCGTTGCAGGTCAGGATACCTGGCTCGAATCTGGGTTTGTGCGCATTGACAGCCTGCTTCGCTGGTGTGCCGACAACCAGATGTATTTGATTCTTGACCTTCATGGAGCTCCCGGCGGCCAGGGACATGATGCCAACATCTCTGACTACGACCCGACAAAACCATCGCTTTGGGAGAGTGCCGAAAACCAACGCAAAACCATTGCCCTGTGGAAAAAACTTGCCCAACGCTATGCCAACGAACAATGGATTGGCGGCTACGACCTCATCAACGAGCCTAACTGGGAGCTTCCCAACGGCAGTCAGCTTCGTCAGCTCTATGTCAGCATCACACAAGCAATCCGACAGGTGGACAACCATCACCTGATCATTATCGAAGGCAACTGGTTTGCCAACGACTATACCGGACTTACTCCGCCATGGGACAACAATATGGTTTATAGTTTTCACAAGTATTGGAATTACAACACTTTGGAATCTATTCAATGGATGATAAACATCCGCGACACCCATTCTGTACCCATTTGGTTGGGCGAATCGGGCGAAAATTCAAATTCATGGTTCACGAGCCTCATTGCACTTTGTGAATCTCAAAAAATCGGATGGTCGTGGTGGCCGGTAAAGAAAGCCGGCATTAACAATGTGCTCATGGTCAACGAAAGTCCTGCCTACAACAATCTGCTCTCTTTTTGGAAGACCGGCCAGCCCCAGATGACCGTACAACAAGCGTTTGACGCTGTTCTTGACTGGGCCGACCGGCATCGCATCGAAAACTGCATGATACAGCGCGATGTGATTGACGCCATGATTCGCCAACCTCACGACAACAGCTCCAGCCCTTTCCGCTTTCATACTACGGCCAATGCCATCAATCTGAGCGACTACGATCTTGGAAAATGTTCGGTAAGCTATTGGGATACCGACACAGCCAATTATCACCTGAACACCGCCTCTTTCACCAATTGGAATCAAGGCTGGAGCTATCGCAACGATGGTGTGGACATCGAAGAAAGCAGTGATACCTTTCCGGGCAGCAATGGTTACAATATCGGCTGGACCGAAGCCGGCGAATGGTTGCAATTTACGGTACATAGCGACAGTGCAGCGGCATATCCTTTGTTGGTTCGCTCGGCCTCGGCATCCATCCCTGCCATCGTACGTTTTGAATGCAACGAAATGGAGATCACACCCAATACAACACTTCCGAATACCGGAGGGTGGCAAAGCTGGGTAGGAAGCCTATTGGAGCCCGTTATCCTGCCGGCAGGCGAAAATAAAATCCGTATCCACTTTGAAAGAGGGGGTTCAAATCTGAGTCTTTTCCGTTTTCTCGACCCTGTGCCTATTGCCAGTGTTGACTTCCATTTCGTTTACGGAAAAACGTTCAACAACAATACCATCCTGCTTACCTTGAATAAAGCCATCACCAATATGGCTTCCAACCCATCCGATTTCGCCCTGAAAATCAATGGTCAACCGGCTCAAATCAATGGCGTTTTTGTGTATCAGGGCAATTCCCACCAGTTGTTATTGAGCATTAATTCCCAATTAAAATATGGCGATCAGATCAGTCTGAGTTATCAGGGCGAAAACATTCTATCGGATAGTCAGGAATTAGAGAGTTTTTCCAATCAGACTATTCAAAATCGTCTTCCAAGGCGGTTTGTGATTCCCACACGCATCGAGGCCGAAGATTTTGACGAAAACAATGGTTTTCAACTTGAAGACTGCACCGATATCGGAGGTGGAAAAAACATTGCTTACGCCAACAATGGCGATTATCTTGATTATCTCATCACGGTTCCCGTTGCAGGTGAATACACTCTTACTTTCCGGCTTGCTTCACTTTATTCAAACGGTCGCATCTCGCTGCGCATTGGCGAAGGCAACCAGTTCACCCCATTAGCCACTCTGAATGTGGCAGCCACAGGCGGTTGGCAAATCTGGAAAAACCAGACCATCAAAGTGCAACTTCCGGCCGGGAACACCACATTCAGGCTTTACTCGCTTGCCGGTGAATACAACATCAACTGGTTTGAATTCAATGGGTTATCAGGAATGAACGACTTTCACGGACTGAAGAATTTAGACATTTACCCCAATCCTACCGAAGGAAATTTCTGGTTGCGTGCCCGGCTCGAGCCTCCCACTGAGCTGACGCTGCGTCTCACCGATCTTCACGGCCGTGAAATAAGCAGCCACATCTTGTCGGAAAACGGTCAGTTTCAGGAGAATATCCGATGCAAACATTGCGCTCCCGGCATTTATTTGCTTCATCTGAGTTCGACACAAGGGAGCACCACGCGAAAGATCGTTTTCCGCTAA

>seed.gene_id_4321

ATGATCAGGTTGGCTGCCCTGCTGATGCTGTTCTGGCCCGCTTCCGCGCTGGCGCTGGAGGTGTCGGGCAACCTGTTGACCGAGAACGGCCAGACCGTGGTGCTGCGCGGCATCGCCATGGGCGATGTCACCGACCTGCCGGCCGAGGTCAATCCCTACCCCGAGATCGCCGGCGACTGGCACGCCAATGCGGTGCGGCTTTCCATCCATCCCGGTACCTGGCGCGACAAGAAGAATGAGGCGCTCGCGACGCTGACGAAGCACGTCGAAGAGGCGCGCGCCGCCGGGCTCTATGTCATCATCGACTATCACGTCATCGGCTTTCCCGACGGCTACGCGCTCGATTATTTCGACGTGACCGAGCCGGACACCAAGACCGACTACTACGATTCGCGCTTCAGCCTGGCGATGGATTTCTGGCTGACCGTGGCGGCGCAGTTCAAGGACCCGGCCATCCTCTACGAATTGTGGAACGAGCCGATCTCCGGCAATGAGGAGGACGAGGGCGTCGGCCTGACCTATTGGAAGAAGTACCGCCCCTATTGGCGGGTGCTGACCGATGCCATCCGCAGCAGCGGCAACAGCAATGTGATGCTGGTGGCCCCGCCGCTCTGGGCCTTCAATGCGCGCGGCCTGACCCAGTCGCTGCTGCCGGACGCGAACACCGCCTATACCTGGCATGTCTATGCCGGCGAGGTGCCCGAGGAATGGGCCGACGCCCTCGACAACCTCGATCGCGAGAAGCCGGTCGTGGTCACCGAATGGGGCTTCGAGCCCGGCGCCAAGCAGCACTGGGCCGGCACCGCCGAGGATTACGGCCAGCCCTTCGCGGCCTTCATGGACCAGCGCGGCCTATCGTCGACCGCCTGGTGCTGGAACCCCGATTACGGCCCCAATCTTCGCCGCGCGGACGGCACGCTCACGGTCTGGGGCAGGTTCGCGAAGGACTACCTGGCGCAGCACGCGCAGTAG

>seed.gene_id_6228

ATGAGTGAGATGAAAAGGAAAGTTTTGCCGGAAGGCTTCTTGTGGGGAGGGGCAGTGGCAGCTAACCAGTTGGAAGGCGGCTGGAACAAGGACGGTAAGGGGGTCAGTGTTGCTGACGTTATGACAGCAGGTGCTCATGGGGTCATGAGGGAGATTACTGAAGGGGTTCTGCCGAATAAATGCTATCCTAACCACGATGCTATAGATTTTTACGGGCATTATAAAGAGGATATTGCTTTGTTTGCAGAAATGGGATTTAAGTGCTTTCGAACAAGCATAGCATGGACACGGATTTTTCCAAATGGGGATGAGCTGCAGCCGAATGAAAAAGGGCTTCAGTTCTATGATGATATGTTTAATGAGCTTTTGAAGTATGGAATTGAGCCGGTAATTACCCTGAGCCATTTTGAAATGCCATATCATTTGGCTAAGGAATATGGCGGGTGGATGAACCGTAAAGTGATTGATTTCTTTGTGAAGTATGCATTGACAGTTATGGAGCGGTATAAGGACAAGGTCAAATACTGGATGACCTTTAATGAAATCAATAATCAAAGAAATGTATCAGCAGATATTTTTGGGTGGACATGTTCGGGAGTTAGATTTTCCGAGTATAAGAATAAGGAAGAAGCAATGTACCAGGTGGTTCATCATCAATTGGTTGCAAGCGCGCTTGTAGTCCAAAAGGGACATGAGATAAATCCGAACTTTAAAATCGGATGCATGTGTTCTTTTGTGCCCGTTTACCCTTATTCCTGCAAGCCGGAAGACGTTATGCTGGCGACAGAATGTATGCATGAAAGATTCTTCTTTGCAGATGTTCATGCAAGGGGGCATTATCCCGGATATGCTCTTAAGGAATGGGAAAGAAAGGGTTATAACATCAAGATGGAACCTGAAGATATCAGTGTATTGCAAAAAGGAAAGGTGGATTTTATAGGTTTCAGTTACTATATGTCTAATGTTGCTAAATCAGATATACATAAGGATGTCAGTAATGCCATGGATGGATCAGCAGAATACTCTGTGCCCAATCCATATATAAAAAGCAGCGGCTGGGGATGGCAGATTGATCCCATAGGTATCAGGTATGCTTTGGTCACTCTATATGAGAGGTATGAGCTGCCGCTGTTTATTGTTGAAAATGGGCTTGGAGCAATTGATGTGATAGAAGAAGACGGAAGCTGCAAGGATGATTATCGTATTGAGTATTTGAAGGCACATATTCAAGAAATGAAAAAAGCAGTTGAATTGGATGGCGTTGATTTGATGGGGTATACTGCATGGGGATGCATTGATTTAGTGTCATTTACAACCGGTGAAATGAAAAAACGCTATGGATTTATTTATGTAGATAAAAACAATGATGGTTCGGGAACTTTGAAGCGGAGCAAAAAGAAATCCTTTGACTGGTATAGGAACGTCATTAAAAGCAACGGAGAGCAGCTGTAA

**Fig. S1.** The nucleotide sequences of 23 glycoside hydrolases. Three target sequences selected in this study are colored in red.


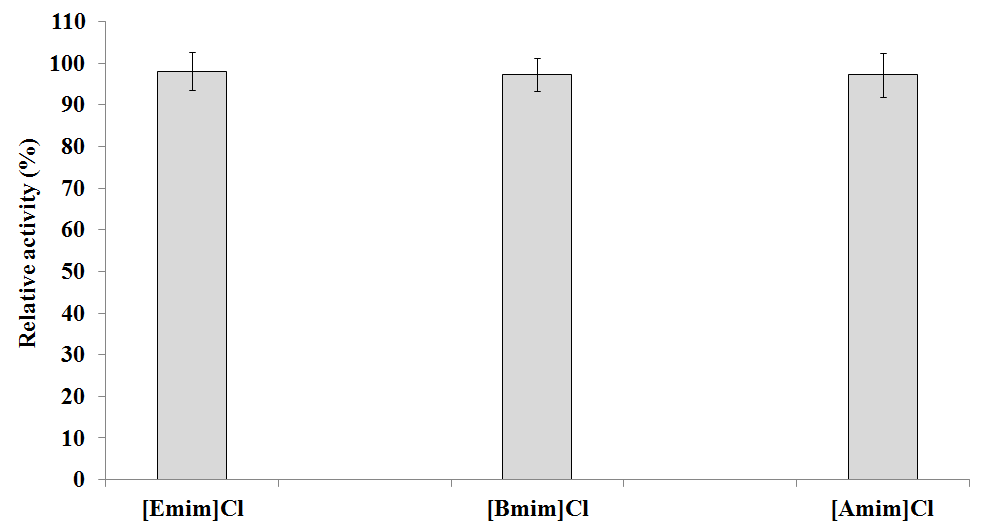


**Fig. S2.** Resistance of the recombinant cel7482 to high concentrations of ILs. The recombinant cel7482 was incubated with CMC at 37°C for 30 min in 50 mM citrate–phosphate buffer (pH 7.0) supplemented with 20% of [Emim]Cl, [Bmim]Cl or [Amim]Cl. The activity in reaction without ILs was set as 100%.

21 49

ch4_3.protein_id_7482 MKKFDGFMAGVNLGGWISQYGKAGKERFDTFITETDIRQIAGWGMDHV**R**L

ch4_1.protein_id_3623 MTNFTGFSAGVNLGGWISQF**W**QKRKEHFDSFITKEDIRQIAGWGMDHV**R**L

*.:* ** ***********: : **:**:***: ***************

93

ch4_3.protein_id_7482 PIDYMVLEDDDRPFEYKEEGFYYVDSCIRWCEKHNLNIILDI**H**RAPGYAF

ch4_1.protein_id_3623 PIDYNILEDDDKPFEYSEEGFSYIDSCIEWCREYGLNIILDL**H**KAPGYAF

**** :*****:****.**** *:****.**.::.******:*:******

102 107 110 141-144

ch4_3.protein_id_7482 Y**F**LN**E**NTLFTDEHMQKRYIGLWRCMAERYKGYGNNLVFEL**LNEL**VEPNSS

ch4_1.protein_id_3623 HSLESNKLFEDNTLQERFINLWKTFAKRYSKYGDNVVFEL**LNEI**VEPNSD

: *:.*.** *: :*:*:*.**: :*:**. **:*:*******:*****.

178

ch4_3.protein_id_7482 RWNNLSKRTVEEIRKIDRNRKIIIGGNLYNSVNTLHELDRIDDDNLVYTF

ch4_1.protein_id_3623 RWNALSKRAVEAIRELDKNRIIIIGGNFYNSVNTLKELDHIDDDRIVYTF

*** ****:** **::*:** ******:*******:***:****.:****

201 204 217 245

ch4_3.protein_id_7482 **H**F**Y**EPHIFTHQKAGWEPLLKDLEVDVTYPSTEETYEVYYP--ERLRQRYL

ch4_1.protein_id_3623 **H**F**Y**LPHLFTHQGASWEKAMSELNFKISYPIGPEDYSKYMQLSESFRRSYQ

*** **:**** *.** :.:*:..::** * *. * * :*: *

ch4_3.protein_id_7482 FGKVVDKEYLRRYMQPALDFARERNVPLYCG**E**YGVIDRAPMDSRLNWHRD

ch4_1.protein_id_3623 ISENMGKDYLREALMPALEFAKERKVAVYCG**E**YGVIDTADMDSRLRWHED

:.: :.*:***. : ***:**:**:*.:********* * *****.**.*

ch4_3.protein_id_7482 LSDLLIEYGIGRAV**W**TYKLMSFPLVDINSAVISDELVKIVSRK-

ch4_1.protein_id_3623 FCSLLIEYGIGRAV**W**SYKLMNFAMVDGNSKVINEDLIKIVSRKI

:..************:****.*.:** ** **.::*:******

**Fig. S3.** Alignment of cel7482 and cel3623 proteins. The amino acid sequences of cel7482 and cel3623 were aligned with ClustalX2.0.12. The identity or similarity of the residues is represented by (*), (:), and (.). The residues in the active site are colored in red. The different residues in the entryway of active site between cel7482 and cel3623 are colored in green and underline.

3PZT ----------------------------------MGSSHHHHHHSSGLVPRGSHMASAAG

id_36 MKKYRCKKKGILWTLVLSLILIMLSGCGETPPEVVSETPLSQQDATSQQPPEAKPEATSE

:..: ::.::. * :: :::

3PZT TKTP--------VAKNGQLSIKGTQLVNRDGKAVQLKGISSHGLQWYGEYVNKDSLKWLR

id_36 PAEPSPESGPTFVERNGNLRVEGTQLVNEAGQPVQLKGVSSHEVASFGYLVTYNALKQMR

. * * :**:* ::******. *:.*****:*** : :* *. ::** :*

3PZT DDWGITVFRAAMYTADG-GYIDNPSVKNKVKEAVEAAKELGIYVIIDWHILNDGNPNQNK

id_36 DDWNLTVFRIAMYTEDATGYIRNPGVKDIVTRIIDDCIDLGIYVIIDWHILYDNTPLKYK

***.:**** **** *. *** **.**: *.. :: . :************ *..* : *

3PZT EKAKEFFKEMSSLYGNTPNVIYEIANEPNG-DVNWKRDIKPYAEEVISVIRKNDPDNIII

id_36 DQAVEFFSEMSARYGDYPNIIYEICNEPNGADTTWEGHIKPYAEEVIPAIRKNDPDNIII

::* ***.***: **: **:****.***** *..*: .*********..***********

3PZT VGTGTWSQDVNDAADDQLKDANVMYALHFYAGTHGQFLRDKANYALSKGAPIFVTEWGTS

id_36 VGTPTWSQDVDIAADDPLPYDNVMYALHFYAGSHGQFLRDKIDYALSKGLPIFVSEWGSC

*** ******: **** * ***********:******** :****** ****:***:.

3PZT DASGNGGVFLDQSREWLKYLDSKTISWVNWNLSDKQESSSALKPGASKTGGWRLSDLSAS

id_36 LNTGDGPTFHEESMEWIKFLDERNISYVNWSFSTKQEGASILRKQIDVNAKWTDVDLTEG

:*:* .* ::* **:*:**.:.**:***.:* ***.:* *: . .. * **: .

3PZT GTFVRENILGT-------------------------------------------------

id_36 GLFAKYAIKGTKDTVLFADGFETKTFGHGKWKRSNDNTTYETENPYKGNYAASIGKDGFL

* *.: * **

3PZT ------------------------------------------------------------

id_36 ERAVTTVPYENLKLHLAYTFVNGKPGDVVRIEWFDGANYNLVTELPLAEEWTELDIALPD

3PZT -----------------------------------

id_36 SASGVHDFSVRITAAVADEDTRLLVDEIWLAAEKK

**Fig. S4.** Alignment of cel36 and 3PZT proteins. The amino acid sequences of cel36 and 3PZT were aligned with ClustalX2.0.12. The identity or similarity of the residues is represented by (*), (:), and (.). The residues in the active site are colored in red.
